# ZEB1 Promotes Alternate Lengthening of Telomeres at Multiple Levels

**DOI:** 10.3390/cancers18030499

**Published:** 2026-02-03

**Authors:** Thomas L. Genetta, J. David Perez-Medero, Hyukjin Jang, Tianpeng Zhang, Braden A. Hussain, James M. Larner

**Affiliations:** Department of Radiation Oncology, University of Virginia Medical Center, 1300 Jefferson Park Ave., Charlottesville, VA 22903, USA; mjg5zf@virginia.edu (J.D.P.-M.); drz5zm@virginia.edu (H.J.); zjv8au@virginia.edu (T.Z.); vnq6tq@virginia.edu (B.A.H.); jml2p@uvahealth.org (J.M.L.)

**Keywords:** Alternate Lengthening of Telomeres, Zinc-Finger E-box-Binding Protein 1, break induced repair, promyelocytic leukemia protein

## Abstract

Background/Objectives: Approximately 10–15% of cancers rely on Alternate Lengthening of Telomeres (ALT), which is a telomerase-independent, BIR (Break-Induced Replication)-based pathway for maintaining long-term replicative potential. Methods: As ALT is over-represented in mesenchymal-type tumors, we investigated, via RNA-seq, the extent to which the EMT-promoting factor ZEB1 regulates this pathway. Results: The ALT-associated genes targeted by ZEB1, including PML, RMI2, POLD4, RPA3 (induced), SLX4, and WRN (repressed), in the aggregate, suggest that it regulates ALT at multiple steps in that pathway. ZEB1-deficient cells showed a significant reduction in telomere length as well as in two hallmarks of ALT, C-circle levels and the size and number of ALT-associated PML Bodies (APBs), which are the telomere-aggregating compartments in which BIR occurs. As one of the most highly regulated genetic targets of ZEB1 was the pro-epithelial alternative splicing factor ESRP1, we investigated whether the repression of this factor was required to generate the PML splice variant isoform IV, which is the major structural component of APBs. We found an inverse relationship between the expression of this protein and levels of PML isoform IV mRNA. Conclusions: These findings suggest a novel role for ZEB1 in promoting ALT both transcriptionally and post-transcriptionally at multiple levels.

## Simple Summary

About 10–15% of cancers–often difficult to treat, pediatric tumors–maintain their telomeres through the Alternate Lengthening of Telomeres (ALT) pathway, the mechanistic details of which are still being elucidated. A significant fraction of these is characteristically mesenchymal–more stem cell-like–in nature, and previous studies have shown a positive correlation between factors that can induce the mesenchymal state and ALT. In this study, we investigated how a pro-mesenchymal gene regulatory protein, called ZEB1, affects ALT mechanistically. We found that it regulates several key factors involved in the initiation, execution and resolution of this pathway, in particular the levels of a protein that forms the major cellular compartment in which ALT is carried out. As ALT is utilized exclusively in cancer cells, identifying potential molecular targets in this pathway offers the possibility of developing efficacious therapies.

## 1. Introduction

The Alternate Lengthening of Telomeres pathway, induced in approximately 10–15% of (usually aggressive) human cancers, is a pathophysiological mechanism of telomere maintenance, making it an attractive therapeutic target [1,2]. Work from many labs over the past several decades has helped elucidate the major mechanisms promoting and driving this pathway, but many details remain to be elucidated [3]. ALT+ cancers, which are over-represented in the pediatric population [4,5,6], display several well-established hallmarks. These include mutations in ATRX/DAXX, the down-regulation of TERT, the presence of Extra Chromosomal Telomeric Repeats (ECTRs, including C-Circles) and a Break-Induced Repair (BIR)-based elongation mechanism that occurs within dedicated, PML IV-based, phase-separated compartments, termed ATL-associated PML Bodies (APBs) comprising scores of factors and multiple telomeres [1,2,3].

Approximately 80% of ALT+ cancers are ATRX/DAXX deficient, leading to a decrease in the deposition of H3.3 in telomeric repeat-embedded nucleosomes, which can promote the de-condensation/euchromatization of this G-rich, constitutively heterochromatic region [7,8]. The consequent increase in transcriptional activity, particularly of the lncRNA TERRA, results in telomeric R-loop formation and displacement of the G-rich DNA strand [9]. This, in turn, can result in an increase in G-quadraplex formation [10]. Individually and together, these structures can amplify the replication stress already inherent in this highly repetitive region with attendant fork collapse and subsequent DNA damage [11,12]. The consequent activation of the DNA Damage Response (DDR) can be triggered through activation of either ATR via RPA-coated ssDNA behind stalled/collapsed replication forks or from resection [13] and/or ATM via the presence of associated Double-Strand Breaks (DSBs) [14]. Although activated ATR both maintains replication fork stability by reducing the association of SLX4 through the phosphorylation of RAD18 [15], and is required for the maintenance of APBs [13], a role for ATM in promoting ALT in animal cells is unresolved [16].

Extra Chromosomal Telomeric Repeats, relatively short, single-stranded repetitive sequences, are a by-product of excessive strand displacement via BLM/POLD [17,18]. Although leading strand-derived G-rich (GGGTTA) repeats in both linear and circular form have been detected, the overwhelmingly predominant form is lagging strand-derived C-circles (CCCTAA repeats), which are a reliable marker for ALT+ cancers [19].

PML-Nuclear bodies (PML-NBs), which play key roles in a broad range of cellular processes [20,21], have been shown to localize to and participate in the repair of DNA-damage induced by IR [22,23], by UV radiation [24] and at telomeres [25]. While much effort is currently focused on defining the molecular details of the recombination-based BIR-dependent pathways that carry out the great proportion of ALT, there is a general consensus that this occurs in PML isoform IV-based NBs, which are termed APBs [25,26,27]. There is substantial evidence showing that the sumoylation-mediated condensation and phase separation of PML IV is required for subsequent recruitment via the SUMO–SIM interactions of ALT-related BIR/DDR client factors [28,29]. A very recent report suggests, however, that the sumoylation of these factors in the absence of PML is sufficient to carry out ALT [30].

The transcription factor ZEB1 (Zinc-Finger E-box Binding Protein) plays a role in the determination/differentiation or regeneration of a number of mammalian tissue compartments, including lymphocyte, bone, adipose, and muscle [31,32,33,34]. Its most well-characterized function is as a driver of the Epithelial-to-Mesenchymal Transition (EMT) in the context of both normal metazoan development and during the progression of carcinomas [35,36]. High ZEB1 (and other EMT-promoting factors) expression is correlated with tumor aggressiveness, metastasis, and the cancer stem cell phenotype [37,38,39]. Recently, several groups, including ours, have demonstrated a role for ZEB1 in the DNA Damage Response as well as in DNA repair itself [40,41,42,43,44,45].

A recent bioinformatics-based analysis of patient samples of later-stage gastric cancers displaying a relaxed chromatin status showed that acquisition of the ALT pathway was clearly correlated with a mesenchymal/“stemy” phenotype and high ZEB1 (and its transcriptional co-activator, YAP1) expression [46]. Given our interest in ZEB1’s role in the DDR [45], and in promoting cancer therapeutic resistance [47,48,49,50], we investigated how and to what extent ZEB1 regulates the ALT pathway. Overall, our data demonstrate that ZEB1 plays a significant role in APB formation and in promoting a RAD52-independent BIR pathway of ALT.

## 2. Materials and Methods

### 2.1. Cell Culture and Generation of ZEB1 Knockout Lines

U2OS and Saos (both ZEB1 proficient and deficient) osteosarcoma and HEK293T cells were all maintained in Dulbecco’s Modified Eagle Medium (DMEM; Gibco, Waltham, MA, USA; Cat.# 11965092) supplemented with 10% FBS and 1% penicillin–streptomycin at 37 °C in a humidified atmosphere containing 5% CO_2_. All cell lines were obtained from ATCC (Gaithersburg, MD, USA) and periodically checked to ensure that they were mycoplasma-free. ZEB1 KO cells were generated via CRISPR/Cas9 genome editing as previously described [45]. Cells were transiently transfected using linear polyethylenimine (PEI, MW = 25,000; Polysciences, Warrington, PA, USA; Cat.# 23966) at a ratio of 1:3 micrograms of DNA to microliters of PEI. For rescue experiments, ZEB1 KO cells were transduced with a lentiviral vector encoding flZEB1 under a constitutive CMV promoter [45].

### 2.2. Telomere Fluorescence In Situ Hybridization (FISH)

For the detection of telomeric DNA, telomere FISH was performed using a AF647-TelC (CCCTAA)-3′ peptide nucleic acid (PNA) probe (PNA Bio, Newbury Park, CA, USA; Cat# F1013). Cells were grown on coverslips, fixed in 4% paraformaldehyde for 10 min, permeabilized with 0.2% Triton X-100, Thermo-Fisher, Waltham, MA, USA; for 15 min, and dehydrated through an ethanol series. Hybridization was performed in 70% formamide and 10 mM Tris-HCl, (Thermo-Fisher, Waltham, MA, USA), pH 7.2, 0.5% blocking reagent (Roche, Basel, Switzerland; Cat# 11096176001) containing 0.3 µg/mL of the PNA probe. Slides were denatured at 80 °C for 10 min and incubated at 4 °C overnight in a humidified chamber. The excess probe was removed via washes in 70% formamide and 10 mM Tris-HCl pH 7.2. DNA was counterstained with DAPI (Thermo-Fisher, Waltham, MA, USA; Cat# 62248), and images were acquired using an Opera Phenix high-throughput confocal microscope (Revvity, Waltham, MA, USA). Fluorescence intensity per nucleus was quantified using Cell Profiler, Version 4.2.8.

### 2.3. Quantitative PCR-Based Absolute Telomere Length (aTL) Assay

Genomic DNA was extracted using the DNeasy Blood & Tissue Kit (Qiagen, Hilden, Germany; Cat# 69504). Telomere length was measured via qPCR as described by O’Callaghan and Fenech [51] with modifications. Reactions were performed using telomere-specific primers and single-copy gene primers for IFNB1. A standard curve was generated from synthetic oligonucleotide standards to calculate absolute telomere length. Reactions were run in triplicate on a Real-Time PCR system (Biorad CFX-96 C-1000 Thermal Cycler, Hercules, CA, USA), and data were analyzed using the ΔΔCt method normalized to IFNB1.

### 2.4. C-Circle Assay

C-circle assays were performed using a modified rolling circle amplification protocol coupled with quantitative PCR detection following the protocol from Henson et al. [52]. Briefly, 30–50 ng of genomic DNA isolated from U2OS cells was incubated in a 20 μL reaction containing 1× ϕ29 DNA polymerase reaction buffer (New England Biolabs (NEB), Ipswich, MA, USA; Cat# B0269SVIAL), 0.25 mM each dATP, dGTP, and dTTP (dCTP omitted), 7.5 U ϕ29 DNA polymerase (NEB Cat# M0269SVIAL), and 0.1 mg/mL BSA. Reactions were carried out at 30 °C for 8 h and then heat-inactivated at 65 °C for 20 min. Amplification products were diluted 1:10 in nuclease-free water and subjected to quantitative PCR using iTaq Universal SYBR Green Supermix (Biorad Cat# 1725121). qPCR reactions (20 μL) used the following telomere-specific primers (forward: 5′-CGGTTTGTTTGGGTTTGGGTTTGGGTTTGGGTTTGGGTT-3′, reverse: 5′-GGCTTGCCTTACCCTTACCCTTACCCTTACCCTACCCT-3′) on a Biorad CFX-96 C-1000 Thermal Cycler with the following cycling conditions: 95 °C, 10 min; 40 cycles of 95 °C, 15 s, 60 °C, 60 s. Standard curves generated from serial dilutions of control telomeric DNA were used to quantify the relative telomeric amplification. A no-polymerase (−Pol) control was included for each DNA sample to account for background amplification. The C-circle signal was defined as the ΔCt between the +Pol and −Pol reactions and normalized to Hemoglobin Subunit Beta (HBB) repeat amplification (housekeeping control primers: Forward 5′-GCTTCTGACACAACTGTGTTCACTAGC-3′ Reverse 5′-CCACCAACTTCATCCACGTTCACC-3′). The fold change in C-circle abundance was calculated relative to wild-type control samples.

### 2.5. ALT-FISH for Detection of C-Rich Extrachromosomal Telomeric Repeats (ECTRs)

To visualize ALT-associated ECTRs, an ALT-FISH protocol was applied using a Alexafluor-647-conjugated (TTAGGG)-3 probe (TelG-A647, PNA Bio Cat# F1014). Cells were fixed and hybridized under non-denaturing conditions to preserve extrachromosomal telomeric DNA. Following hybridization, samples were washed, counterstained with DAPI, and imaged by confocal microscopy. The number of foci per nucleus was quantified from maximum intensity projections using Cell Profiler, and foci were defined by consistent intensity and size criteria across all conditions.

### 2.6. Immuno-FISH for ALT-Associated PML Bodies (APBs)

To assess APBs, immuno-FISH was performed by combining immunostaining for PML with telomere FISH, following the protocol of Cesare et. al. [53]. Cells were treated with pre-extraction buffer, fixed and blocked, and then incubated with rabbit anti-PML antibody (1:1000, Santa Cruz, Dallas, TX, USA; sc-966) followed by anti-mouse Alexa Fluor 488-conjugated secondary antibody (Invitrogen, Thermo-Fisher, Waltham, MA, USA; Cat# A21200). Subsequently, FISH was carried out using an AF647-labeled TelC probe (PNA Bio) as above. Confocal z-stacks were captured using an Opera Phenix high-throughput confocal microscope, and co-localization analysis between PML and telomeric signals was performed using Cell Profiler. At least 50 nuclei were analyzed per condition.

### 2.7. PML Promoter Mutagenesis

A human PML promoter fragment (1300 bp including the transcription start site) fused upstream of a secreted version of the Gaussia luciferase (gLuc) cDNA was purchased from Genecopoeia, Rockville, MD, USA; (Cat # HPRM44457-LvPG04). For normalization between experimental conditions, this construct also harbored a constitutively active second reporter cDNA: secreted alkaline phosphatase (SEAP). Site-specific mutations were carried out via Q5 mutagenesis (New England Biolabs Cat # E0554S) using the following primer pairs: MCAT/TEAD site: For 5′-ATCACTGGTACACAGGAGCCAGCACAGTGGTTGCGATGG, Rev 5′-GTACTCTGTTCCAACTTAGAAACAGACTAGAAAGG; Upstream/Distal Ebox: For 5′-ATGTATGAACTGTGCCCCTTTCCCCTTACCAGCTGG, Rev 5′-ACCTCACTGGGTCAGCGCCCCGGC.

### 2.8. Luciferase Assays

The wt and mutant reporter constructs were transfected into the indicated cell types using PEI as above, and 48 h later, aliquots of the culture media were removed for analysis of both gLuc and SEAP in a 96-well plate format using a BioTek Synergy plate reader, Winooski, VT, USA, according to the manufacturer’s instructions (Genecopoeia Cat # LF031).

### 2.9. ChIP Analysis

Chromatin Immunoprecipitation and subsequent real-time qPCR assays were performed as previously described [45]. Two microliters of ChIP-isolated DNA was amplified on a Bio-Rad CFX-96 Real-Time PCR Machine using iTaq Universal SYBR Green Supermix (Bio-Rad) with the following cycling parameters: 95 °C, 3 min 1×; 95 °C, 5 s, 60 °C, 20 s, 40×; followed by a melt curve of 65–95 °Cwith 0.5 °C increments. A 132 bp fragment incorporating the MCAT/TEAD binding site was amplified using the primer pair: For, 5′-GGAAGTTGCGTATCTGCC, Rev, 5′-GCTCCTGTGTACCCATTCC. Results, presented as percent of input, were derived from at least three independent experimental runs with each qPCR analysis performed in triplicate, and the results were averaged with SEM.

### 2.10. TaqMan-Based qRT-PCR

The total RNA from 10^6^ cells/condition was isolated, and the quality was assessed as described in Section 2.11 above. First-strand cDNA library synthesis was carried out using SuperScript IV VILO Master Mix (Invitrogen Cat# 11756050), including a second DNAse treatment, according to the manufacturer’s instructions. A minus reverse transcriptase (RT) control was included for all experimental conditions. For the real-time PCR, a 20 μL reaction vol included 2 μL of a 1:100 dilution of the cDNA transcriptome libraries and 10 μL of 2× PrimeTime Gene Expression Master Mix (IDT, Coralville, IA, USA; Cat# 1055770). In the case of PML IV, 10 μM of each For and Rev primer and 5 μM of a FAM-labeled probe (IDT Assay ID: Hs.PT.58.26680167) was included in the reaction. For interrogating total PML isoform mRNA levels, 1 μL of a combination 20× Primer/FAM-labeled Probe set spanning exons 2–3 (IDT) was used. From our RNA-Seq data, the housekeeping gene succinate dehydrogenase, subunit A (SDHA) showed virtually no change in mRNA levels between ZEB1-proficient and deficient U2OS cells, and a combination 20× Primer/SUN-labeled Probe set (IDT Assay ID: Hs.PT.58.40170798) to interrogate the levels of this message was employed for normalization. Real-time TaqMan PCR reactions and detection was performed on a BioRad CFX-96 C-1000 Thermal Cycler using cycling parameters: 95 °C, 3 min, 1×; then 95 °C, 5 s, 60 °C, 30 s, for 40 cycles. Four experimental replicates were analyzed per condition, including minus RT and negative (water) controls, and each analysis was carried out at least three times. The qCt values for all samples were averaged, and the relative fold change (normalized to SDHA), compared to controls, in total or isoform IV PML mRNA levels was calculated using the ΔΔCt method. Primer sequences used for this analysis: Pan-PML, For: 5′-GGCACCCGCAAGACCAA, Rev: 5′-ACTGTGGCTGCTGTCAAG, Probe: 5′-/56-FAM/ATCTACTGC/ZEN/CGAGGATGTTCCAAGC/3IABkFQ/-3′; PML IV, For: 5′-CAGCTCGGAAGACTCAGATGC, Rev: 5′-CCAGGAGAACCCACTTTCATTG, Probe: 5′-/56-FAM/ACAGCAGCA/ZEN/GTGAGTCCAGTGACCTCC/3IABkFQ/-3′; SDHA, For: 5′-GGAGTGCCGTGGTGTCA; Rev: 5′-TGGTGTGGGCAGACGTG; Probe: 5′-/5SUN/TGCTCTTAT/ZEN/GCGATGGATGGACCC/3IABkFQ/-3′.

### 2.11. RNA-Seq

The isolation of total RNA from 10^6^ cells/condition, including an RNAse-free DNAse step to reduce/eliminate genomic DNA, was carried out using a Qiagen Total Mini Kit (Cat# 74104), according to the manufacturer’s instructions. All samples had an RNA Integrity Number equivalent (RIN*^e^*) number of 10 (Agilent RNA TapeStation, Santa Clara, CA, USA).

### 2.12. Bioinformatics

All bioinformatics analysis was carried out by the UVA Bioinformatics Core Facility. The bioinformatics data derived from the RNA-seq data, including heat maps, volcano plot, principal component analysis, etc.) along with the accompanying source code can be accessed in Supplementary File S1.

### 2.13. Western Blot

Whole-cell lysates were prepared in RIPA buffer supplemented with protease and phosphatase inhibitors (Roche, Basel, Switzerland; Cat# 11836170001). Protein concentration was determined by BCA assay (Thermo/Pierce Cat# 23227). Samples (10–20 µg per lane) were resolved on 4–16% SDS-PAGE gels and transferred to PVDF membranes. Membranes were blocked in 5% BSA for 1 h and incubated o/n at 4 °C with primary antibodies: anti-ZEB1, 1:1000 (Invitrogen PA5-28221); anti-PML, 1:1000 (Santa Cruz Biotech. sc-966, Dallas, TX, USA); anti-GAPDH, 1:2000 (Santa Cruz Biotec. sc-44724); anti-SDHA, 1:30,000 (Proteintech, Rosemont, IL, USA; 66588-1-Ig). After washing (TBS-0.2%/Tween 20, 3X), membranes were incubated with LiCor secondary antibodies (1:5000; Cat# 926-68070 and 936-32211) for 1 h RT and washed again. Blots were scanned with LiCor Odyssey CLx imager (LiCor Bio, Lincoln, NE, USA), and band intensity was quantified using FIJI (Version 2.0.0-rc-68/1.53n).

### 2.14. Statistical Analysis

All quantitative experiments were conducted with at least three independent biological replicates unless otherwise specified. Data are presented as mean ± standard error of the mean (SEM). Statistical significance was assessed using an unpaired two-tailed Student’s *t*-test or one-way ANOVA with Tukey’s post hoc test for multiple comparisons. *p*-values less than 0.05 were considered statistically significant. Graphs and analyses were generated using GraphPad Prism version 10 (GraphPad Software San Diego, CA, USA).

## 3. Results

### 3.1. ZEB1 Regulates ALT-Associated Genes

**Figure 1:**
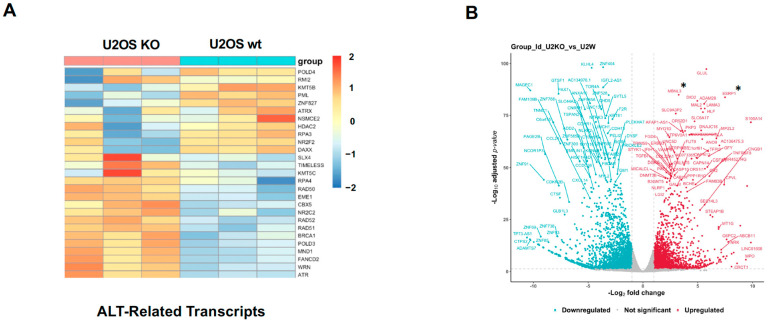
ZEB1 regulates a number of ALT-associated genes. (**A**) Heatmap showing that steady-state levels of RNA for several key players in the ALT pathway, including POLD3, POLD4, SLX4, and PML are targets of ZEB1. (**B**) Volcano plot depicting fold-changes of ZEB1 target genes. The splicing regulators, MBNL3 and ESRP1, denoted by asterixis, are among the most significantly induced transcripts in ZEB1 KO U2OS cells. RNA-Seq Supporting Data is shown in Figure S1 and File S2.

A recent bioinformatics-based report demonstrated a correlation between ZEB1-driven EMT and the ALT pathway in gastric cancer [46]. Given this, and, as part of our ongoing efforts to characterize ZEB1’s role in DNA metabolism, we performed an RNA-Seq analysis on total RNA isolated from ZEB1-proficient vs. deficient U2OS cells. As U2OS cells employ the ALT pathway [54,55], we analyzed the differential expression of genes that are known to play a critical role in the implementation or regulation of this pathway. The loss of ZEB1 resulted in a reduction in a subset of several key drivers of this pathway, including PML [29], SLX4 [56] and RMI2 [57] (Figure 1A). Other genes encoding ALT-specific regulators such as POLD4, ATRX and DAXX were induced, suggesting that ZEB1 may promote a particular branch or sub-pathway of ALT (though U2OS cells have been shown to harbor functionally inactive mutations in the case of the ALT-inhibitor ATRX [12]).

Among the most highly-repressed ZEB1 targets—denoted by asterixis in the Volcano Plot in Figure 1B—are two regulators of pre-mRNA alternative splicing (AS), Epithelial Splicing Regulatory Protein 1 (ESRP1, a “Master Regulator” of the Epithelial phenotype [58,59,60]), and Muscle Blind-Like Splicing Regulator 3 (MBNL3, which, together with ESRP1, promotes the splicing of muscle-specific structural components) [61,62]. The downregulation of pro-epithelial splicing patterns/protein isoforms would be consistent with a pro-ALT mesenchymal phenotype [46]. These results prompted us to ask whether the ZEB1-mediated regulation of AS in this way might also play a role in ALT (see below).

### 3.2. ZEB1 Is Critical for Telomere Maintenance via ALT

**Figure 2:**
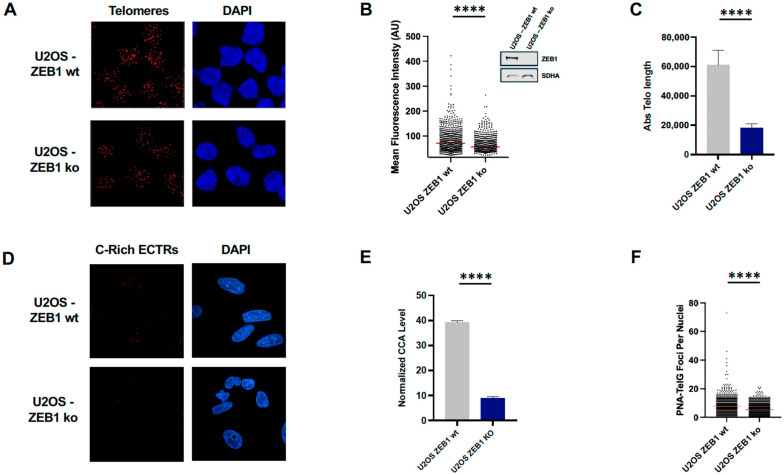
ZEB1 deficiency impairs telomere length, maintenance, and ALT activity in U2OS cells. (**A**) Representative fluorescent in situ hybridization (FISH) images using a TEL-C probe to visualize telomeric DNA (red foci) in ZEB1-proficient (wt) and ZEB1KO U2OS cells. (**B**) Quantification of mean fluorescence intensity of telomeric foci shown in (**A**), showing a reduced telomere signal in ZEB1 KO cells. Inset, WB showing ZEB1 protein depletion in ZEB1 ko U2OS cells, SDHA, succinate dehydrogenase, loading control. (**C**) Quantitative PCR-based measurement of absolute telomere length, revealing a significant reduction in telomere length in ZEB1 KO cells. (**D**) Representative result of ALT-FISH detection of C-rich extra-chromosomal telomeric repeats (ECTRs), visualized using a TEL-G-Alexa-Fluor 647 probe, in ZEB wt vs. KO cells. (**E**) qPCR-based quantification of C-circle abundance (C-Circle Assay) relative to a single-copy gene, shows dramatically reduced ALT activity in ZEB1 KO U2OS cells. (**F**) Quantification of C-rich ECTR foci per nucleus, showing significantly fewer ALT-associated foci in ZEB1 KO cells. Data represent the mean ± SEM; **** *p* < 0.0001. The whole blots (uncropped blots) are shown in Figure S2.

Given the RNA-Seq results, we first asked to what extent ZEB1 affects ALT by assessing a number of well-established hallmarks of that pathway. We first measured directly ZEB1’s effect on ALT-dependent telomere length in ZEB1-proficient (CRISPR-non-targeted) vs. CRISPR-depleted (Knock-Out/KO) U2OS cells. ZEB1 loss results in a significant reduction in both the fluorescence intensity (2-fold) of FISH-labeled telomeres (Figure 2A,B) with a 3-fold reduction in absolute telomere length determined via qPCR (Figure 2C). Relative levels of a signature characteristic of the ALT pathway, Extra-Chromosomal Telomeric Repeat (ECTR) elements [3,12,63]—in this case, C-circles—dramatically reduced in the absence of ZEB1 (Figure 2D) as measured by either qPCR (Figure 2E) or ALT-FISH (Figure 2F).

### 3.3. ZEB1 Regulates Size and Number of ALT-Associated PML Bodies (APBs)

**Figure 3:**
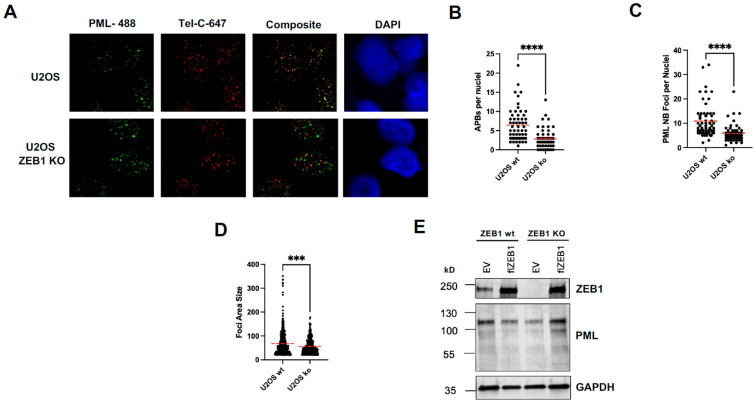
ZEB1 regulates APB levels in U2OS cells. (**A**) Immuno-FISH (IF-FISH) showing the co-localization of PML nuclear bodies (PML-NBs, green) with telomeres (TelC, red) in U2OS wt and ZEB1 KO cells. The composite image shows merged red and green signals. (**B**) Quantification of ALT-associated PML bodies (APBs), defined as TelC and PML co-localized foci per nucleus, demonstrating reduced APBs in ZEB1 KO cells. (**C**) Quantification of total PML-NBs per nucleus, showing significantly fewer nuclear foci in ZEB1 KO cells. (**D**) Measurement of PML-NB foci size (area), indicating a decrease in average foci size in the absence of ZEB1. (**E**) Western blot analysis of ZEB1 and PML protein expression in U2OS wild-type and ZEB1 knockout cells, with or without ectopic ZEB1 (flZEB1), GAPDH served as a loading control. ZEB1 knockout reduces PML levels, which are rescued by ZEB1 re-expression. Data represent the mean ± SEM; **** *p* < 0.0001, *** *p* < 0.001. The whole blots (uncropped blots) are shown in Figure S2.

ALT is carried out in APBs, which are dynamic, liquid-phase separated compartments that aggregate/concentrate both telomeric DNA and scores of proteins/factors involved in strand invasion and subsequent HR-based replication [3]. The major structural component of APBs is the PML protein—predominately isoform IV [28,29]. Interestingly, our RNA-Seq results show that the steady-state levels of total PML transcripts are reduced in ZEB KO cells (Figure 1A). As the loss of ZEB1 results in a reduction in both telomere length and ALT-associated ECTRs (Figure 2), we were next prompted to ask whether APBs were likewise affected. Compared with their ZEB1-proficient counterparts, ZEB1 KO U2OS cells had one half the number of APBs/nucleus (Figure 3A,B). Additionally, the total number of PML nuclear bodies (either ALT- or non-ALT-associated) were significantly reduced in both number and size (Figure 3C,D). Next, using an antibody that recognizes a common epitope near the amino terminus of all six predominant PML isoforms, mirroring the results from the RNA-seq data, we saw a 2-fold reduction in total cellular PML protein levels in ZEB1-deficient (KO) cells compared to ZEB1-proficient counterparts. While the over-expression of flZEB1 cDNA appeared to have no effect on endogenous PML protein levels in ZEB1-proficient cells, in ZEB1 KO cells, the PML protein levels were restored to greater than normal wt levels (Figure 3E).

### 3.4. ZEB1 Transcriptionally Activates the PML Gene

**Figure 4:**
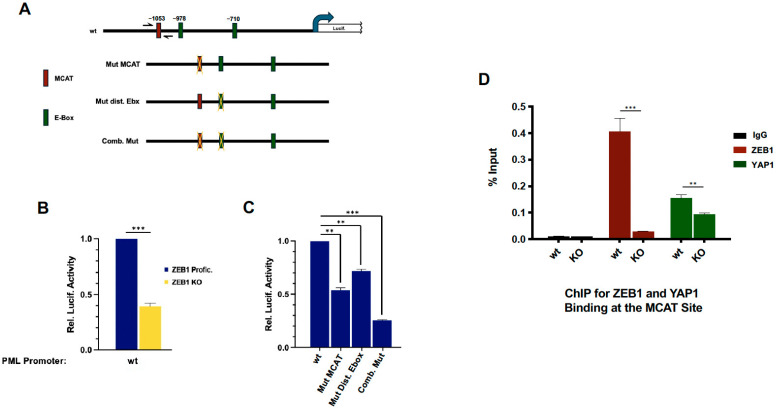
PML is transcriptionally induced by ZEB1. (**A**) Top, schematic representation of a 1300 bp fragment of the human PML promoter fused upstream a luciferase reporter construct with an MCAT binding site (−1053 bp from the start site, red box) and two canonical ZEB1 E-box binding sites (−978 bp and −710 bp, green boxes); the mutations (denoted by yellow crosses) employed in the subsequent assays are shown below. (**B**) Compared with wt, PML promoter activity is reduced by 60% in ZEB1 KO cells. (**C**) Mutating the MCAT site alone results in a loss of nearly half with the nearby distal Ebox mutation on its own reducing luciferase activity by about one third, and together, by about 75% of wt. (**D**) Using the primers denoted by the blue arrows in panel (**A**), ChIP assays show that the MCAT site binds a complex that includes both ZEB1 and YAP transcription factors. Data represent the mean ± SEM; *** *p* < 0.001, ** *p*< 0.01.

Given that ZEB1-depleted cells showed a reduction in both APB size and number (Figure 3), and that our RNA-Seq results and protein analysis show that PML is a ZEB1-targeted gene (Figure 1A and Figure 3E,F), we next asked whether this is a result of direct transcriptional regulation. The proximal 1000 bp of the human PML promoter region harbors six potential ZEB1-binding sites (E-boxes, CANNTG/A [64,65,66]), including two preferred, “canonical”, sites, CAGGTG. In addition, this sequence contains a TEAD/MCAT binding site [67], which, in a cooperative fashion with a nearby E-box, has previously been shown to localize a TEAD/YAP/ZEB1 heterotrimeric transcriptional activation complex (Figure 4A; [68]). Compared to its activity in ZEB1-proficient U2OS cells, a luciferase reporter construct driven by the wt PML promoter sequence showed an approximately 60% reduction in the absence of ZEB1 (U2OS KO cells), which is consistent with our previous results (Figure 4B, yellow bar). Mutating either the MCAT site or the proximal downstream E-box site individually resulted in a 50% and 30% reduction in luciferase activity, respectively, while the combination of the two reduced it by nearly 80% (Figure 4C).

To demonstrate that ZEB1 regulated the PML promoter directly, we carried out a standard ChIP assay, using PCR primers spanning the MCAT site (Figure 4A). A robust ZEB1-mediated amplification of this PCR product was virtually eliminated in ZEB1 U2OS KO cells, while a less pronounced effect in both ZEB1-proficient and deficient cells was obtained with this same primer pair using an antibody against YAP1 (Figure 4D).

### 3.5. Levels of PML Isoform IV Are Reduced 2-Fold in ZEB1 KO Cells

**Figure 5:**
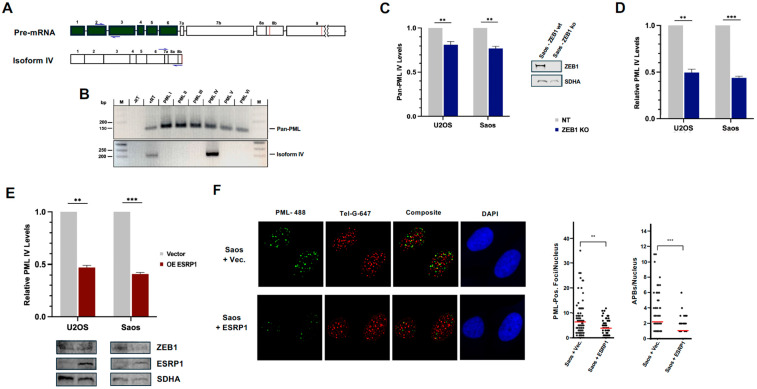
ZEB1 regulates alternative splicing of PML pre-MRNA through ESRP1. (**A**) Top: Schematic of the PML pre-mRNA, exons common to all prominent isoforms (I–VI) are highlighted in green. Primers crossing intron 2, used to detect all common isoforms [“Pan-PML” in panel **B**], are denoted by blue arrows; Bottom: Schematic for Isoform IV; primers specific for this isoform are shown in blue. (**B**) PCR primers (blue arrows in panel **A**) used in the RT-PCR analyses can distinguish PML isoform IV from the other prominent isoforms. PCR products visualized on a 3% agarose gel with ethidium bromide staining. Top panel: The total RNA isolated from U2OS cells was used in an RT-PCR reaction using the Pan-PML primers with (+RT) or without (-RT) reverse transcriptase. In separate PCR reactions using these same primers, individual expression plasmids encoding each of the six prominent isoforms were used as the template; the primers specific for isoform IV amplified only that cognate template (Bottom panel). (**C**,**D**) In both U2OS and Saos cells, ZEB1 deficiency results in a 20% reduction in total PML (Pan-PML) mRNA levels but a greater than 50% loss of PML IV levels; WB for ZEB1 protein levels in the Saos cells is shown between panels (**C**,**D**), while ZEB1 protein levels in U2OS cells are shown Figure 2B; SDHA is the loading control. (**E**) Stable over-expression of a cDNA for ESRP1 or empty lentiviral vector (V) in ZEB-proficient (wt) U2OS or Saos cells phenocopies the loss of PML IV seen in their ZEB1 KO counterparts: below, WB showing ZEB1 and ESRP1 protein levels in the four cell lines; above, SDHA, loading control. (**F**) Representative IF-FISH analysis demonstrates that a reduction in PML IV via the over-expression of ESRP1 in ZEB1-proficient (wt) Saos cells (panel **E**) results in an overall reduction in PML bodies (green/AlexaFluor-488) and about a 50% reduction in APBs (Composite panel; quantified at right); telomeres are visualized by AlexaFluor-647-PNA FISH probe (red panel). Data represent the mean ± SEM; *** *p* < 0.001, ** *p*< 0.01. The whole blots (uncropped blots) are shown in Figure S2.

PML isoform IV, the predominant and key structural component of APBs, is one of six major alternative splicing (AS) products derived from the PML pre-mRNA [20,69]. Our RNA-seq data showed that the second and third most highly expressed transcripts in ZEB1-depleted cells were, respectively, ESRP1, a well-characterized master regulator of pro-epithelial AS [59,60], and MBNL3, which regulates AS specifically in dividing muscle precursor cells during normal skeletal muscle cell determination/differentiation [61,62] (see Volcano Plot, Figure 1B). As the majority of cancers that utilize the ALT pathway are both phenotypically and genetically mesenchymal (as are the U2OS and Saos osteosarcoma cell lines employed in this study [54,55]), we were prompted to investigate whether PML itself might be subject to AS regulation by ESRP1. To address this question, we first measured, via RT-PCR, the relative levels of PML RNA using primers spanning exons 2 and 3, which are common to all PML isoforms [69,70] (Figure 5A, top). Compared to ZEB1-proficient cells, the total PML RNA levels declined over 20% in both ZEB1-deficient osteosarcoma cell lines (Figure 5C). Most interestingly, primers specific for isoform IV (Figure 5A,B) showed a 50% decline again in both cell lines (Figure 5D).

### 3.6. Over-Expression of the Pro-Epithelial Splicing Factor ESRP1 in ZEB1-Proficient Cells Reduces Levels of PML Isoform IV

ZEB1’s role as an EMT-promoting factor and its transcriptional down-regulation of pro-epithelial splicing patterns via the repression of ESRP1/2 are both well documented [71,72]. Given the above results, including the significant induction of ESRP1 mRNA in ZEB1 KO cells (Figure 1B), we next asked whether one function of ZEB1 in the context of an ALT-permissive cellular environment might be to promote a pro-ALT splicing pattern for PML. To this end, we stably over-expressed fl human ESRP1 (or empty Lentiviral vector) in either ZEB1-proficient (i.e., normal) U2OS or Saos cells and performed RT-PCR analysis on isolated RNA as above. In both cell types, compared to vector controls, ESRP1 over-expression resulted in a reduction in PML IV mRNA to levels slightly lower than we observed in ZEB1- or Saos-deficient cells (Figure 5E). Furthermore, regarding an associated reduction in APBs in these cells (Figure 5F), the phenocopying levels seen in ZEB1-deficient U2OS cells (Figure 3) support a role for pro-mesenchymal/anti-epithelial splicing in ALT.

## 4. Discussion

There are several well-documented events that can promote the transition of cancer cells to the ALT pathway. The order and extent of acquisition of these is likely tumor-specific and can include a loss of function of ATRX/DAXX for H3.3 nucleosome deposition, down-regulation of TERT, replicative stress, mesenchymal phenotype, and progression toward metastasis [1,2,3,12,46]. As a well-documented driver of EMT, metastasis, and the stem cell phenotype [36,37,38,39], ZEB1 has also been shown to impose replication stress [41,42,43,44]. It follows that as ATRX-mutated cancer cells progress toward later, more aggressive stages, ZEB1 protein levels are induced, inflicting an even greater burden of replication stress, further amplifying ALT-permissive conditions.

This study, however, reveals a broader, more direct role for ZEB1 in ALT. Our RNA-Seq data show that ZEB1 up-regulated a subset of key ALT-promoting genes, including PML, POLD4, the BTR complex component RMI2, and RPA3, while down-regulating several inhibitors/attenuators, such as TERT and TIMELESS. Interestingly, other known ALT effectors, such as POLD3, RAD51 and RAD52 are modestly repressed, while certain inhibitors, such as chromatin modifiers ATRX (which is functionally inactive in both U2OS and Saos cells [12]) and DAXX are induced by ZEB1 (Figure 1). This particular group of ALT-related genes targeted by ZEB1 may reflect the up-regulation or reliance on a specific sub-pathway of ALT or that the U2OS cell line is frozen phenotypically along the progression toward total reliance on the ALT pathway. Further work is will be required to understand how ZEB1 and/or other EMT-inducing factors regulate ALT-related factors at the protein level.

U2OS cells deficient in ZEB1 show a clear, significant loss in telomere length (Figure 2B,C) as well as a reduction in APB size and number (Figure 3B–D). The concurrent reduction in a signature hallmark of ALT, C-circles (Figure 2E), is consistent with these data. As our RNA-Seq results show that ZEB1 down-regulates both RAD51 and RAD52, in the aggregate, these data suggest that (1) ZEB1 may be promoting a RAD 52-independent BIR pathway [56], (2) it is acting upstream of this pathway at the genetic level, and (3) it is affecting ALT-associated factors at both the transcriptional and post-transcriptional (via AS) levels (although we cannot rule out the possibility that ZEB1 and/or other EMT-promoting factors may play an additional role in this process at telomeres or APBs).

The effect of ZEB1 expression on another well-studied component of ALT-associated BIR, the fate of the progressively elongating repetitive sequences, either through dissolution (via the BTR complex) or resolution (via SLX4/SLX4IP complex) is not entirely clear, and it is based solely on the RNA-seq data. The competition and regulatory crosstalk (mediated, in part, by SLX4IP) between these mutually exclusive pathways is essential for the telomere maintenance via ALT [73]. While SLX4 is slightly repressed, SLX4IP mRNA is induced, and a clearer understanding of the role of ZEB1 on this dynamic and in ALT in general must await an analysis of its effects on ALT effectors/regulators at the protein level.

The formation of APBs is initiated through the coalescence of PML IV, forming dynamic, BIR-dedicated, phase separated compartments with telomere ends and HR/BIR-related factors as client proteins. The initial trigger for this aggregation appears to be the SUMOylation of both telomere-associated proteins (e.g., shelterin components [74]) and many of the key effectors of ALT, such as RAD51, RAD52, POL3, POL4, BLM, etc. [28]. PML isoform IV, the major structural component of APBs, is itself SUMOylated and harbors SUMO-interaction motifs (SIMS), which help to localize and concentrate these replication-related factors [29,75], although a recent report by Zhao et. al. suggests that the sumoylation of ALT-mediated factors in the absence of PML is sufficient for ALT [30].

We show here that ZEB1 transcriptionally regulates several key players involved in both the establishment of APBs and effectors of this pathway. Most interestingly, the major structural component of APBs, PML, is not only a direct transcriptional target of ZEB1 (Figure 1A and Figure 4) but appears also to be regulated at the level of alternative splicing through the repression, by ZEB1, of a “master regulator” of epithelial-specific AS, ESRP1 [58,60] (Figure 5E).

In both normal mammalian development and in solid tumor progression, cells undergoing an EMT will, via transcription factors such as ZEB1, SNAIL, and SLUG, repress ESRP1 and its co-splicing regulator ESRP2, resulting in an overall “mesenchymal” splicing pattern [60,72]. Given our RT-PCR results with PML mRNA in the mesenchymal U2OS cell line, it is reasonable to hypothesize that as tumor cells progress through an EMT to a more aggressive, pro-metastatic state, one consequence of this transition might be to promote the AS of PML pre-mRNA to a pro-ALT pattern (via the down-regulation of ESRP1) to attain threshold levels of isoform IV.

In summary, our data strongly support a role for the metastasis-promoting factor ZEB1 in driving ALT not only by imposing replicative stress but by genetically regulating a number of ALT-related factors, including the key APB structural component PML IV. Among the future goals based on this work will be to determine whether ESRP1 acts on PML mRNA directly or indirectly and the extent to which other EMT factors promote the ALT pathway.

## Data Availability

All original data used to generate the findings in this report are available can be obtained upon reasonable request to the corresponding author. The bioinformatics data derived from the RNA-seq analysis, (including raw sequence scores, heat maps, volcano plot, principal component analysis, etc.) along with the accompanying source code are freely available online as referenced in Section 2.12.

## Supplementary Materials

The following supporting information can be downloaded at https://www.mdpi.com/article/10.3390/cancers18030499/s1, Figure S1: RNA-Seq Supporting Data. Figure S2: The whole blot (uncropped blots) Data. File S1: The most salient Bioinformatics Data. File S2: The raw transcript counts for both U2OS ZEB1 wt vs. ZEB1 KO and HEK293T ZEB1 wt vs. ZEB1 KO.
