# Supplementary material for: ZEB1 Promotes Alternate Lengthening of Telomeres at Multiple Levels
Source: Cancers (Basel). 2026 Feb 3;18(3):499. doi: 10.3390/cancers18030499 (PMC12897190; doi:10.3390/cancers18030499)
Supplement: Supplementary file 1 [file cancers-18-00499-s001.zip › cancers-4083380 Figure S1.pdf]

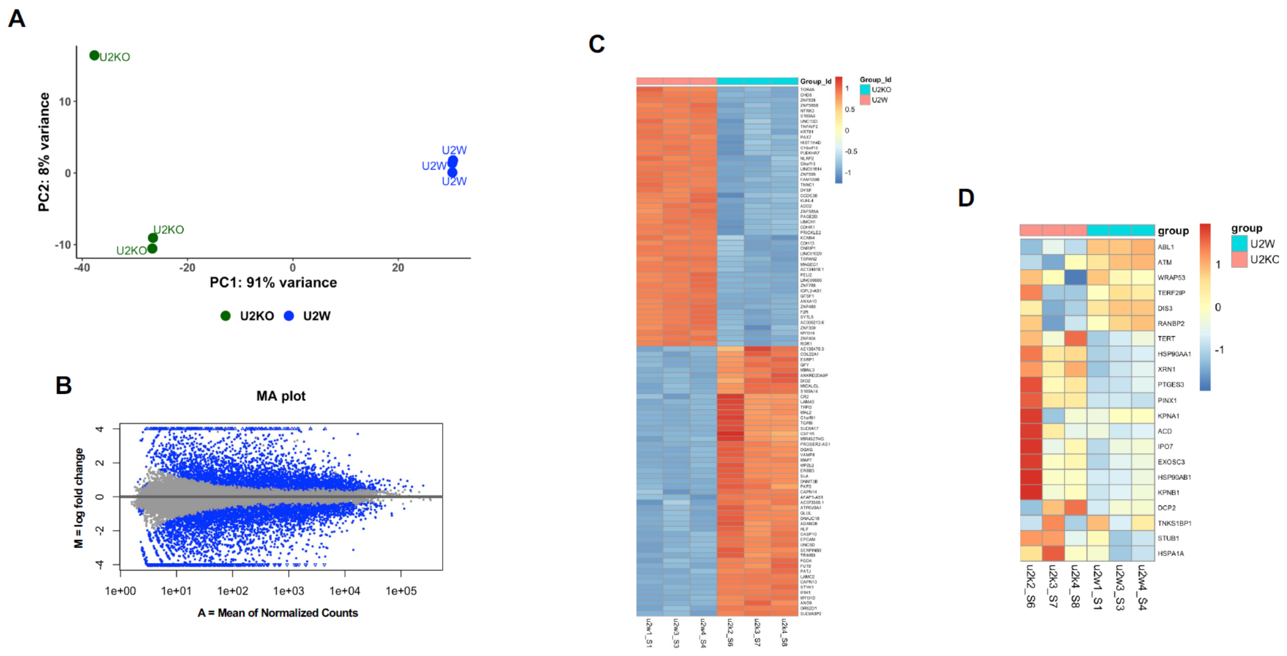

Figure S1. Key Bioinformatics data and link to website for complete analysis. (A) Principal Component Analysis of the data shows that ZEB1 expression accounts for 91% of the total variance between the control vs experimental groups (PC1), with clustering among the two groups showing good consistency between experimental replicates (PC2); (B) the MA plot, shows the effect of ZEB1 depletion on gene expression levels on the Y axis (M = log2 (ZEB1 KO/ZEB1 wt), vs. mean log-intensity of overall gene expression on the XAxis (A = [log2 (ZEB1 KO) + log2 (ZEB1wt)]/2). The funnel-shaped symmetry around M= 0 (the data is centered along the X-axis) indicates that the data are of high enough quality without the need for normalization to correct for treatment/reagent or handling effects; (C) A heat map showing the 50 most highly-induced and the 50 most highly-repressed genes as a result of ZEB1 depletion; (D) heat map showing the effect of ZEB1 depletion on genes involved in the telomerase-dependent telomere elongation pathway. Complete raw data files for gene expression analysis, further supporting statistical analysis and all software files are available in Supplementary File S2.
