# Supplementary material for: ZEB1 Promotes Alternate Lengthening of Telomeres at Multiple Levels
Source: Cancers (Basel). 2026 Feb 3;18(3):499. doi: 10.3390/cancers18030499 (PMC12897190; doi:10.3390/cancers18030499)
Supplement: Supplementary file 1 [file cancers-18-00499-s001.zip › cancers-4083380 Figure S2 WB Source Data .pdf]

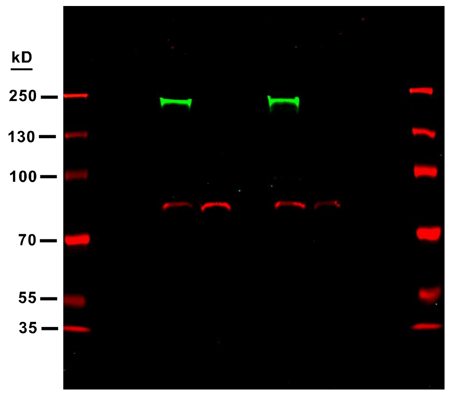

ZEB1 / SDHA

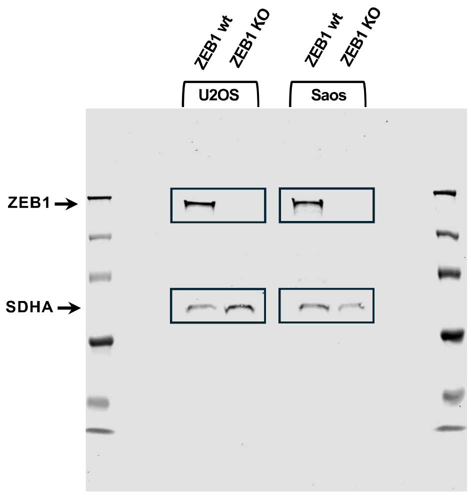

	U2OS		Saos	
ZEB1	18304.9	0	21339.7	0
SDHA	9298.5	14202.9	12175	5552.9
ZEB1 Status:	wt	KO	wt	KO

Western Blot referring to Figure 2 and Figure 5; SDHA = hSuccinate Dehydrogenase

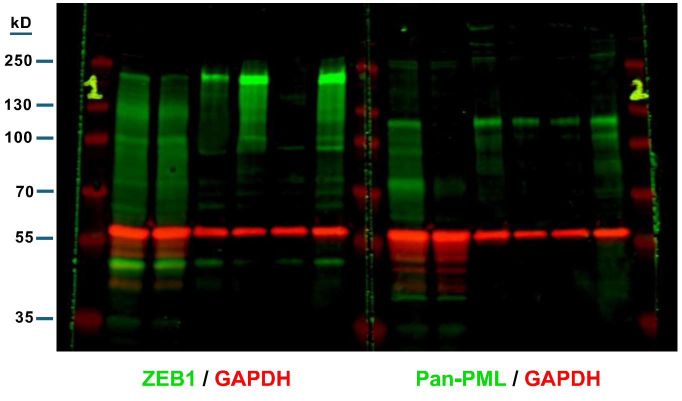

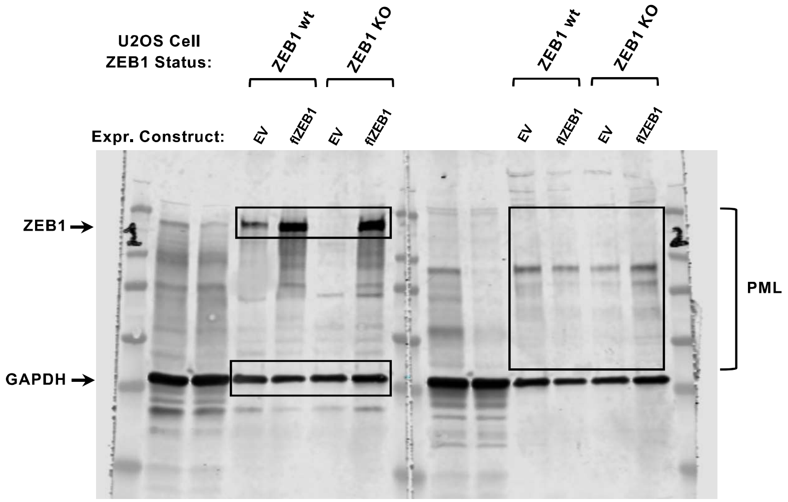

	EV	flZEB1	EV	flZEB1
ZEB1 →	15368.85	28566.37	2981.10	29740.08
GAPDH →	22249.23	19062.93	22640.64	28032.93
ZEB1 Normalized to GAPDH	0.69	1.50	0.13	1.06

	EV	flZEB1	EV	flZEB1
Pan-PML →	21272.97	10824.13	9294.49	27498.44
GAPDH →	32087.08	21660.62	26696.37	32781.69
PML Normalized to GAPDH	0.66	0.50	0.35	0.84

Western Blot referring to Figure 3; GAPDH = hGlyceraldehyde Dehydrogenase

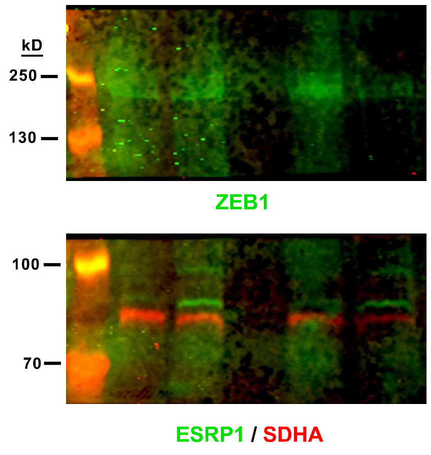

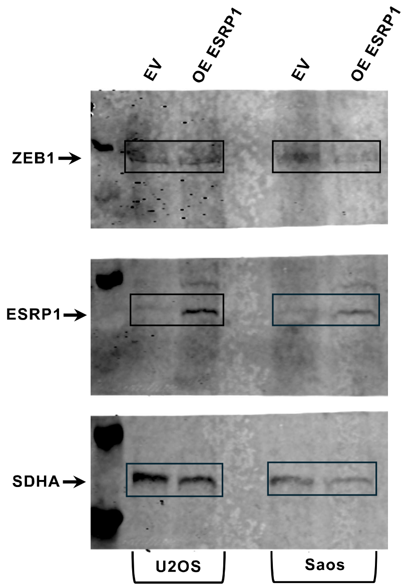

	U2OS		Saos	
	EV	OE ESRP1	EV	OE ESRP1
ZEB1 →	27116	25281.1	22527.1	6521
ESRP1 →	17862	31535.4	12960.5	12998.3
SDHA →	27167.8	19465.3	11642.8	6009
ZEB1 Normalized to SDHA →	1	1.3	1.9	1.1
ESRP1 Normalized to SDHA →	0.7	1.6	1.1	2.2

Western Blot referring to Figure 5E; SDHA = hSuccinate Dehydrogenase
