# Supplementary material for: ZEB1 Promotes Alternate Lengthening of Telomeres at Multiple Levels
Source: Cancers (Basel). 2026 Feb 3;18(3):499. doi: 10.3390/cancers18030499 (PMC12897190; doi:10.3390/cancers18030499)
Supplement: Supplementary file 1 [file cancers-18-00499-s001.zip › cancers-4083380 File S1.pdf]

R Notebook

Code

DGE analysis of contrast U2KO Vs U2W

Hide

```
library("apeglm")
#library( "DESeq" ) #This is correct
library("DESeq2") #This is correct
library("tidyverse")
library("ggrepel")
library("ggplot2")
```

Hide

```


library(ggpubr)
library("ggvenn")
library("VennDiagram")

library(readxl)
library("xlsx")
```

Hide

```
library(clusterProfiler)
library(ggplotify)
library(enrichplot)
library(forcats)
```

Hide

```
#if (!requireNamespace("BiocManager", quietly = TRUE))
#    install.packages("BiocManager")

#BiocManager::install("fgsea")
#library(fgsea)

#install.packages("msigdbr")
library(msigdbr)

library(ggfortify)
```

Hide

```
#library(topGO)
#library(GO.db)
library(biomaRt)
library(Rgraphviz)
```

Load the count matrix

Hide

```
count_matrix <- read.table("/Volumes/UVABX-PK/LarnerLab_Tom_M617TG5561LarnerRNAS16p_scRNASeq_Nov_2024/FASTQ/LarnerLab_Tom_M617TG5561LarnerRNAS16p_RNASeq_Nov_2024_Gene_Count_Table.txt", header=TRUE, row.names=1)

count_matrix
```

	X29k1_S13 <dbl>	X29k2_S14 <dbl>	X29k3_S15 <dbl>	X29k4_S16 <dbl>	X29w1_S9 <dbl>	X29w2_S10 <dbl>	X29w3_S11 <dbl>	X29w4_S12 <dbl>	u2k1_S5 <dbl>
TSPAN6	7042	7971	6521	6751	7214	6421	5943	6568	0
TNMD	4	13	1	5	3	5	5	3	0
DPM1	2381	2690	2324	2399	2402	1935	1690	2056	2355
SCYL3	1129	1100	1162	1057	871	797	662	694	517
C1orf112	2910	3341	2837	2809	2642	2212	2071	2067	1682
FGR	1	0	0	3	1	2	0	4	2
CFH	0	0	1	1	1	11	1	0	0
FUCA2	3017	3597	2728	2928	3304	3400	2925	3243	4605
GCLC	3113	3527	3087	3181	4046	3697	3299	3437	4118
NFYA	5255	5788	5237	5025	5440	5089	4355	4786	2646

1-10 of 56,609 rows | 1-10 of 16 columns Previous 1 2 3 4 5 6 ... 100 Next

Load the meta data

Hide

```
metadata <- read.table("/Volumes/UVABX-PK/LarnerLab_Tom_M617TG5561LarnerRNAS16p_scRNASeq_Nov_2024/FASTQ/LarnerLab
_Tom_M617TG5561LarnerRNAS16p_RNASeq_Nov_2024_metadata.txt", header = TRUE, sep = ",")

metadata
```

SampleName <chr>	Group_Id <chr>
X29k1_S13	29KO
X29k2_S14	29KO
X29k3_S15	29KO
X29k4_S16	29KO
X29w1_S9	29W
X29w2_S10	29W
X29w3_S11	29W
X29w4_S12	29W
u2k1_S5	U2KO
u2k2_S6	U2KO

1-10 of 16 rows Previous 1 2 Next

Hide

```
NA
```

displaying sample groups based on group id

Hide

```
table(metadata$Group_Id)
```

```

29KO  29W U2KO  U2W
   4    4    4    4
```

Starting differential gene expression analysis

Contrast U2KO Vs U2W

Denominator is U2W

Hide

```
# measure the effect of treatment, U2Wling for sample level differences

dds <- DESeqDataSetFromMatrix(count_matrix, metadata, ~Group_Id)
```

```
converting counts to integer mode
Warning in DESeqDataSet(se, design = design, ignoreRank) :
  some variables in design formula are characters, converting to factors
```

Hide

```
dds$Group_Id <- relevel(dds$Group_Id , "U2W")
idx <- rowSums( counts(dds) >= 5) >= 4 # atleast 4 samples (out of 16 here) have value greater than or equal to 5
dds <- dds[idx,]
dds <- DESeq(dds)
```

```
estimating size factors
estimating dispersions
gene-wise dispersion estimates
mean-dispersion relationship
final dispersion estimates
fitting model and testing
```

Hide

```
# Write out normalized counts
library(tidyverse)
dds %>% counts(normalized=TRUE) %>% write.csv("/Volumes/UVABX-PK/UVABX-PK-temp/BIOINFORMATICS-CORE/LarnerLab_Tom_
M617TG5561LarnerRNAS16p_RNASeq_Nov_2024/LarnerLab_Tom_M617TG5561LarnerRNAS16p_RNASeq_Nov_2024_Gene_Count_Table-tx
i-normcounts.txt",row.names = TRUE)
```

PCA Plot

Hide

```
##################PCA plot#################
#rld = rlog(dds, blind = FALSE)
rld = vst(dds, blind = FALSE)
library(ggrepel)
library(ggplot2)
plotpca <- plotPCA(rld, intgroup = c("Group_Id"), returnData = TRUE)
```

```
using ntop=500 top features by variance
```

Hide

```
percentVar <- round(100 * attr(plotpca, "percentVar"))
ggplot(data = plotpca, aes(PC1, PC2, color=metadata$Group_Id)) + geom_point(aes(colour = metadata$Group_Id),size=
4) +
  geom_text_repel(aes(PC1, PC2, label = metadata$Group_Id), max.overlaps = 40) +   xlab(paste0("PC1: ",percentVar
[1],"% variance")) +
  ylab(paste0("PC2: ",percentVar[2],"% variance")) +  scale_color_manual(values = c("darkgreen","blue","black","r
ed","burlywood4","cadetblue","chocolate4", "cyan", "aquamarine", "darkgray", "deeppink", "azure4", "darkolivegree
n1")) + theme_bw() + theme(panel.border = element_blank(), panel.grid.major = element_blank(),
panel.grid.minor = element_blank(), axis.line = element_line(colour = "black")) + theme( axis.title.x = element_t
ext(color="black", size=14, face="bold"), axis.title.y = element_text(color="black", size=14, face="bold")) + the
me(legend.text = element_text(color = "black", size = 12, face="bold")) + theme(legend.title=element_blank()) + t
heme(text = element_text(size=14, face="bold")) +  theme(legend.position="bottom")
```

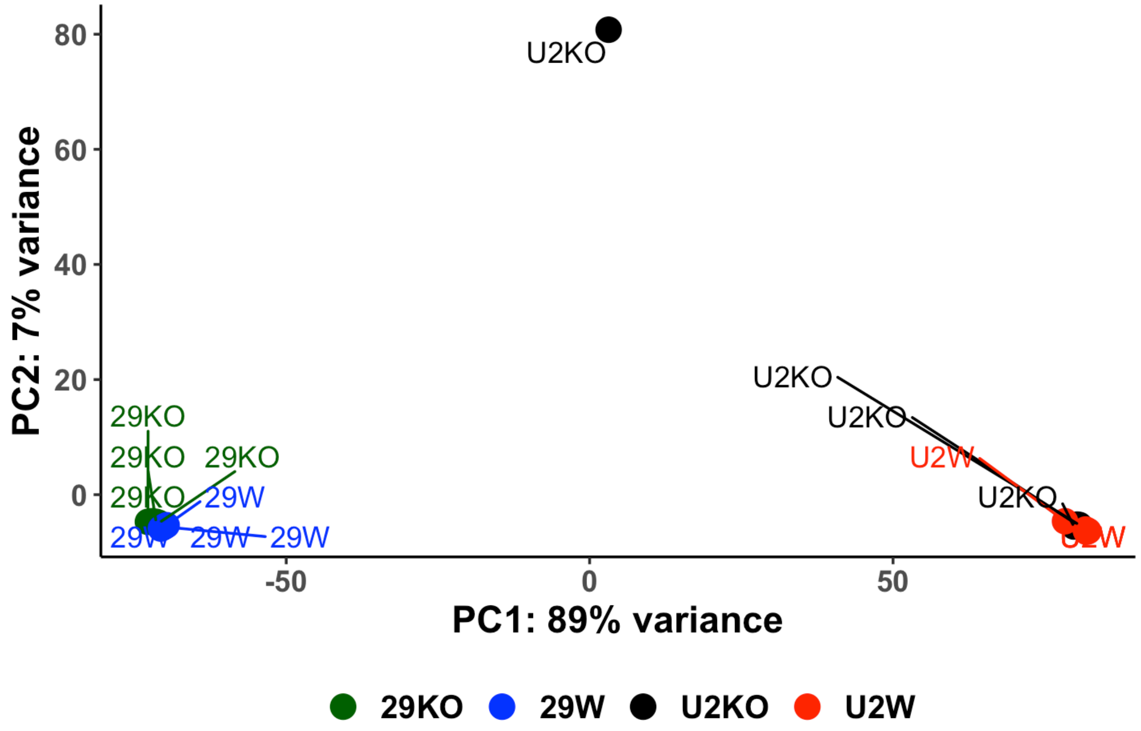

Subsetting the samples based on celltype

Hide

```
count_matrix<-count_matrix[,c(9:16)]

metadata<-metadata[c(9:16),]

metadata
```

	SampleName <chr>	Group_Id <chr>
9	u2k1_S5	U2KO
10	u2k2_S6	U2KO
11	u2k3_S7	U2KO
12	u2k4_S8	U2KO
13	u2w1_S1	U2W
14	u2w2_S2	U2W
15	u2w3_S3	U2W
16	u2w4_S4	U2W

8 rows

Starting differential gene expression analysis

Contrast U2KO Vs U2W

Denominator is U2W

Hide

```
# measure the effect of treatment, U2Wling for sample level differences

dds <- DESeqDataSetFromMatrix(count_matrix, metadata, ~Group_Id)
```

```
converting counts to integer mode
Warning in DESeqDataSet(se, design = design, ignoreRank) :
  some variables in design formula are characters, converting to factors
```

Hide

```
dds$Group_Id <- relevel(dds$Group_Id , "U2W")
idx <- rowSums( counts(dds) >= 5) >= 2 # atleast 2 samples (out of 8 samples here) have value greater than or equ
al to 5
dds <- dds[idx,]
dds <- DESeq(dds)
```

```
estimating size factors
estimating dispersions
gene-wise dispersion estimates
mean-dispersion relationship
final dispersion estimates
fitting model and testing
```

PCA Plot

Hide

```
#################PCA plot#################
#rld = rlog(dds, blind = FALSE)
rld = vst(dds, blind = FALSE)
library(ggrepel)
library(ggplot2)
plotpca <- plotPCA(rld, intgroup = c("Group_Id"), returnData = TRUE)
```

```
using ntop=500 top features by variance
```

Hide

```
percentVar <- round(100 * attr(plotpca, "percentVar"))
ggplot(data = plotpca, aes(PC1, PC2, color=metadata$Group_Id)) + geom_point(aes(colour = metadata$Group_Id),size=
4) +
  geom_text_repel(aes(PC1, PC2, label = metadata$Group_Id), max.overlaps = 40) +   xlab(paste0("PC1: ",percentVar
[1],"% variance")) +
  ylab(paste0("PC2: ",percentVar[2],"% variance")) +  scale_color_manual(values = c("darkgreen","blue","black","r
ed","burlywood4","cadetblue","chocolate4", "cyan", "aquamarine", "darkgray", "deeppink", "azure4", "darkolivegree
n1")) + theme_bw() + theme(panel.border = element_blank(), panel.grid.major = element_blank(),
panel.grid.minor = element_blank(), axis.line = element_line(colour = "black")) + theme( axis.title.x = element_t
ext(color="black", size=14, face="bold"), axis.title.y = element_text(color="black", size=14, face="bold")) + the
me(legend.text = element_text(color = "black", size = 12, face="bold")) + theme(legend.title=element_blank()) + t
heme(text = element_text(size=14, face="bold")) +  theme(legend.position="bottom")
```

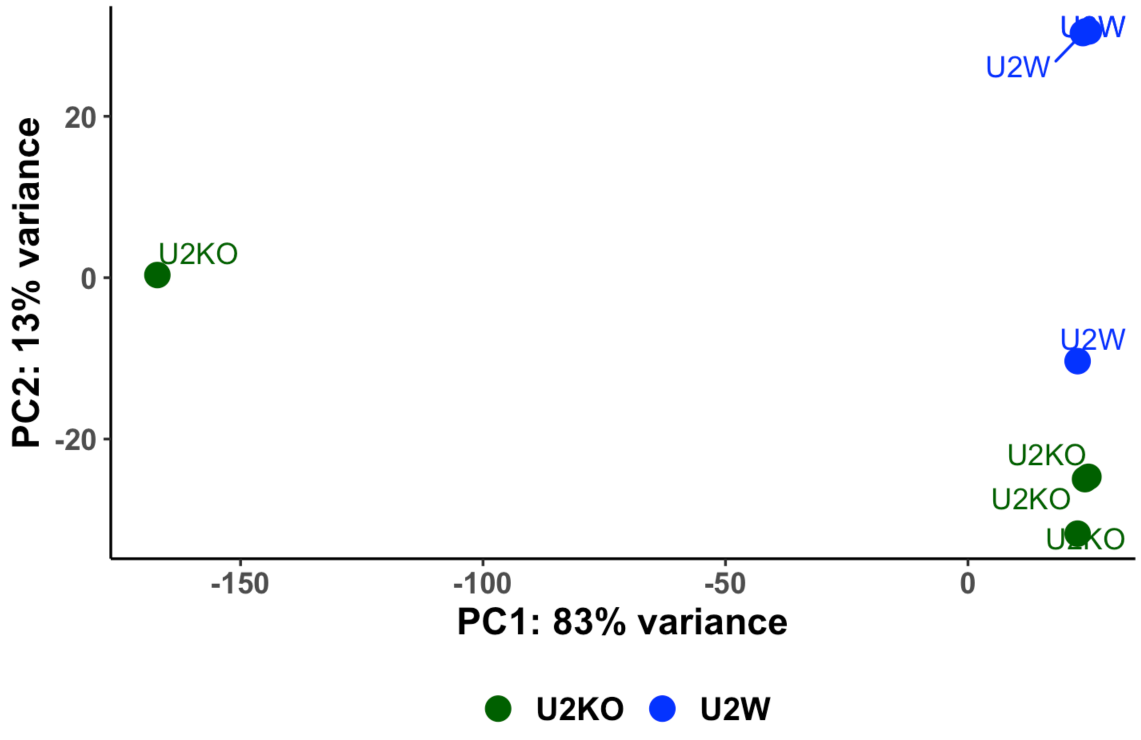

Hide

```
##################PCA plot#################
#rld = rlog(dds, blind = FALSE)
rld = vst(dds, blind = FALSE)
library(ggrepel)
library(ggplot2)
plotpca <- plotPCA(rld, intgroup = c("Group_Id"), returnData = TRUE)
```

```
using ntop=500 top features by variance
```

Hide

```
percentVar <- round(100 * attr(plotpca, "percentVar"))
ggplot(data = plotpca, aes(PC1, PC2, color=metadata$Group_Id)) + geom_point(aes(colour = metadata$Group_Id),size=
4) +
  geom_text_repel(aes(PC1, PC2, label = metadata$SampleName), max.overlaps = 40) +   xlab(paste0("PC1: ",percentV
ar[1],"% variance")) +
  ylab(paste0("PC2: ",percentVar[2],"% variance")) +  scale_color_manual(values = c("darkgreen","blue","black","r
ed","burlywood4","cadetblue","chocolate4", "cyan", "aquamarine", "darkgray", "deeppink", "azure4", "darkolivegree
n1")) + theme_bw() + theme(panel.border = element_blank(), panel.grid.major = element_blank(),
panel.grid.minor = element_blank(), axis.line = element_line(colour = "black")) + theme( axis.title.x = element_t
ext(color="black", size=14, face="bold"), axis.title.y = element_text(color="black", size=14, face="bold")) + the
me(legend.text = element_text(color = "black", size = 12, face="bold")) + theme(legend.title=element_blank()) + t
heme(text = element_text(size=14, face="bold")) +  theme(legend.position="bottom")
```

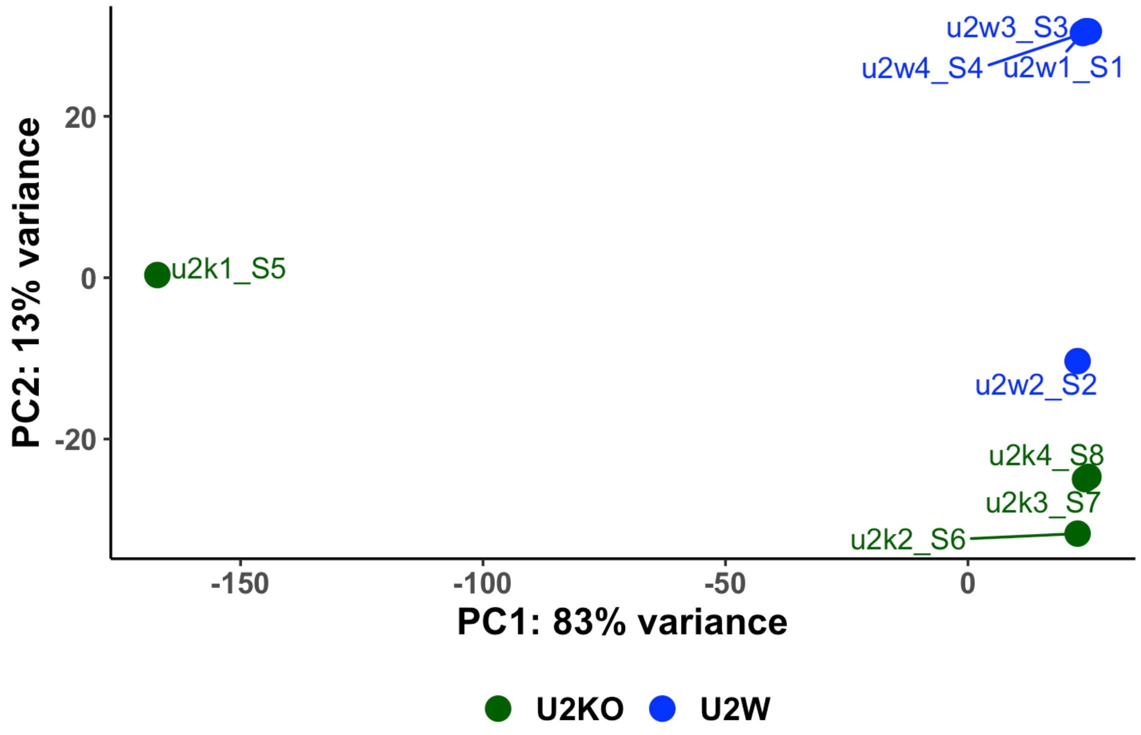

Removing two samples based on PCA (u2k1_S5,u2w_S2)

Subsetting the samples based on celltype

Hide

```
count_matrix<-count_matrix[,c(2,3,4,5,7,8)]

metadata<-metadata[c(2,3,4,5,7,8),]

metadata
```

	SampleName <chr>	Group_Id <chr>
10	u2k2_S6	U2KO
11	u2k3_S7	U2KO
12	u2k4_S8	U2KO
13	u2w1_S1	U2W
15	u2w3_S3	U2W
16	u2w4_S4	U2W

6 rows

Starting differential gene expression analysis

Contrast U2KO Vs U2W

Denominator is U2W

Hide

```
# measure the effect of treatment, U2Wling for sample level differences

dds <- DESeqDataSetFromMatrix(count_matrix, metadata, ~Group_Id)
```

```
converting counts to integer mode
Warning in DESeqDataSet(se, design = design, ignoreRank) :
  some variables in design formula are characters, converting to factors
```

Hide

```
dds$Group_Id <- relevel(dds$Group_Id , "U2W")
idx <- rowSums( counts(dds) >= 5) >= 2 # atleast 2 samples (out of 8 samples here) have value greater than or equ
al to 5
dds <- dds[idx,]
dds <- DESeq(dds)
```

```
estimating size factors
estimating dispersions
gene-wise dispersion estimates
mean-dispersion relationship
final dispersion estimates
fitting model and testing
```

PCA Plot

Hide

```
##################PCA plot#################
#rld = rlog(dds, blind = FALSE)
rld = vst(dds, blind = FALSE)
library(ggrepel)
library(ggplot2)
plotpca <- plotPCA(rld, intgroup = c("Group_Id"), returnData = TRUE)
```

```
using ntop=500 top features by variance
```

Hide

```
percentVar <- round(100 * attr(plotpca, "percentVar"))
ggplot(data = plotpca, aes(PC1, PC2, color=metadata$Group_Id)) + geom_point(aes(colour = metadata$Group_Id),size=
4) +
  geom_text_repel(aes(PC1, PC2, label = metadata$Group_Id), max.overlaps = 40) +   xlab(paste0("PC1: ",percentVar
[1],"% variance")) +
  ylab(paste0("PC2: ",percentVar[2],"% variance")) +  scale_color_manual(values = c("darkgreen","blue","black","r
ed","burlywood4","cadetblue","chocolate4", "cyan", "aquamarine", "darkgray", "deeppink", "azure4", "darkolivegree
n1")) + theme_bw() + theme(panel.border = element_blank(), panel.grid.major = element_blank(),
panel.grid.minor = element_blank(), axis.line = element_line(colour = "black")) + theme( axis.title.x = element_t
ext(color="black", size=14, face="bold"), axis.title.y = element_text(color="black", size=14, face="bold")) + the
me(legend.text = element_text(color = "black", size = 12, face="bold")) + theme(legend.title=element_blank()) + t
heme(text = element_text(size=14, face="bold")) +  theme(legend.position="bottom")
```

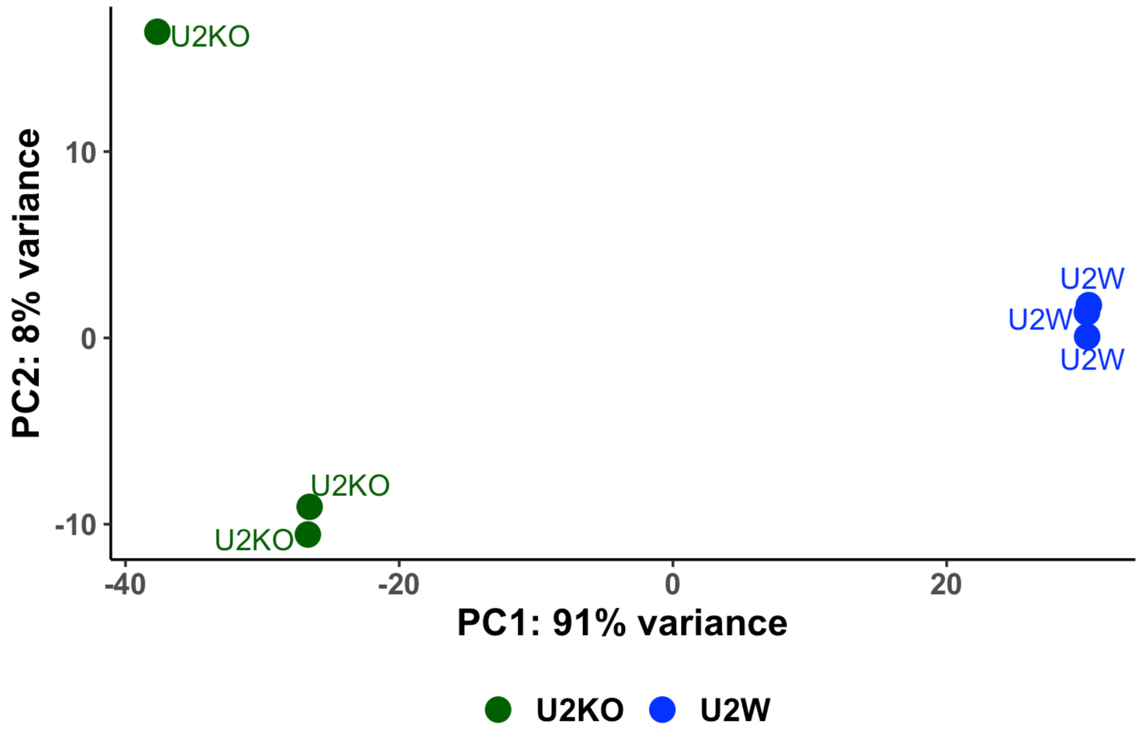

Hide

```
#################PCA plot################
#rld = rlog(dds, blind = FALSE)
rld = vst(dds, blind = FALSE)
library(ggrepel)
library(ggplot2)
plotpca <- plotPCA(rld, intgroup = c("Group_Id"), returnData = TRUE)
```

```
using ntop=500 top features by variance
```

Hide

```
percentVar <- round(100 * attr(plotpca, "percentVar"))
ggplot(data = plotpca, aes(PC1, PC2, color=metadata$Group_Id)) + geom_point(aes(colour = metadata$Group_Id),size=4) +
  geom_text_repel(aes(PC1, PC2, label = metadata$SampleName), max.overlaps = 40) +   xlab(paste0("PC1: ",percentVar[1],"% variance")) +
  ylab(paste0("PC2: ",percentVar[2],"% variance")) +  scale_color_manual(values = c("darkgreen","blue","black","red","burlywood4","cadetblue","chocolate4", "cyan", "aquamarine", "darkgray", "deeppink", "azure4", "darkolivegreen1")) + theme_bw() + theme(panel.border = element_blank(), panel.grid.major = element_blank(),
panel.grid.minor = element_blank(), axis.line = element_line(colour = "black")) + theme( axis.title.x = element_text(color="black", size=14, face="bold"), axis.title.y = element_text(color="black", size=14, face="bold")) + theme(legend.text = element_text(color = "black", size = 12, face="bold")) + theme(legend.title=element_blank()) + theme(text = element_text(size=14, face="bold")) +  theme(legend.position="bottom")
```

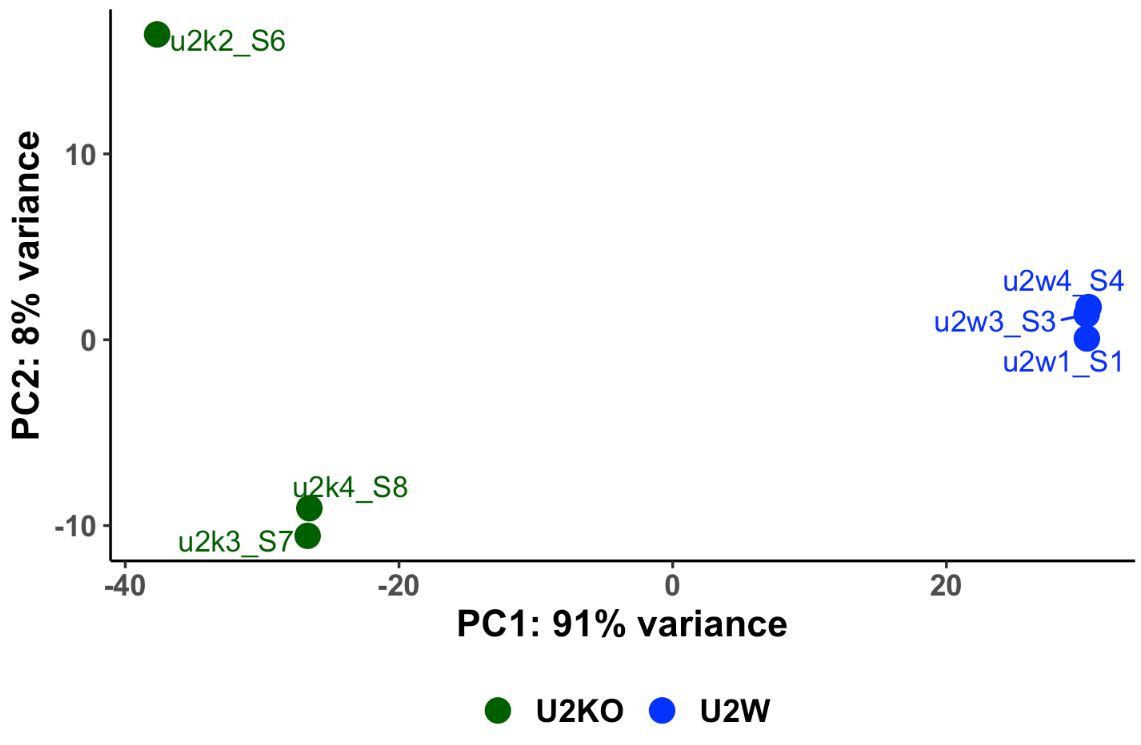

MA Plot: Contrast U2KO vs U2W (Group_Id_U2KO_vs_U2W)

Hide

```
library("apeglm")
resultsNames(dds)
```

```
[1] "Intercept"            "Group_Id_U2KO_vs_U2W"
```

Hide

```
res <- results(dds, name="Group_Id_U2KO_vs_U2W", tidy = TRUE)
resLFC <- lfcShrink(dds, coef="Group_Id_U2KO_vs_U2W", type="apeglm")
```

```
using 'apeglm' for LFC shrinkage. If used in published research, please cite:
    Zhu, A., Ibrahim, J.G., Love, M.I. (2018) Heavy-tailed prior distributions for
    sequence count data: removing the noise and preserving large differences.
    Bioinformatics. https://doi.org/10.1093/bioinformatics/bty895
```

Hide

```
plotMA(resLFC, xlab = "mean of normalized counts", alpha = 0.05, main = "MA plot", ylim=c(-4,4))
```

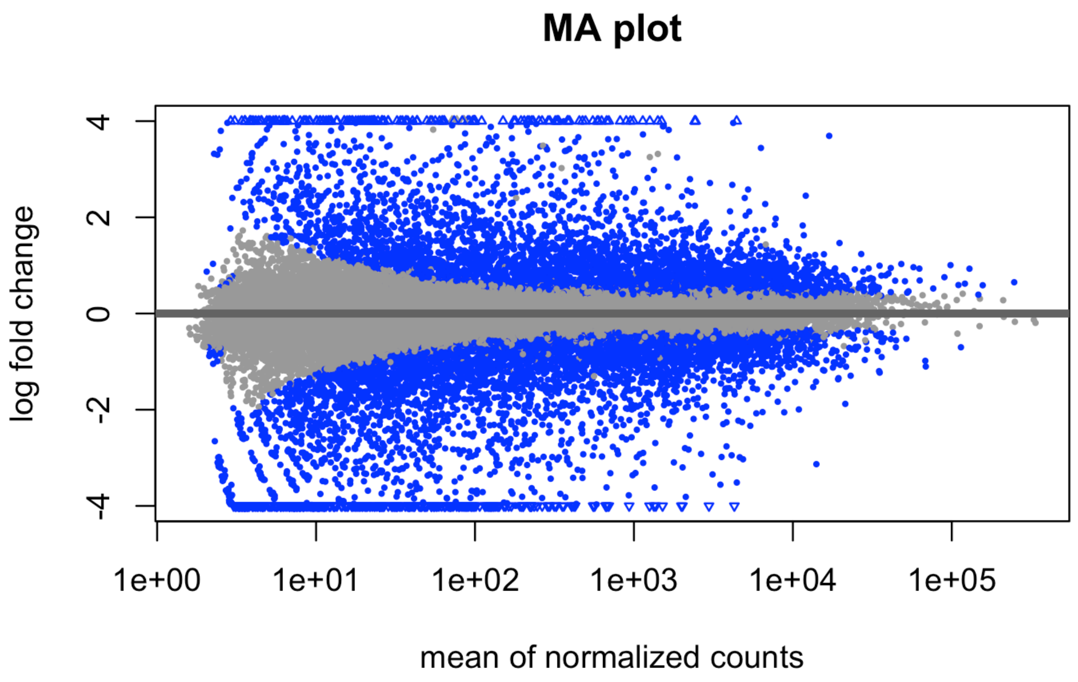

Hide

```
#write files for data needed for volcano plots
write.table(res, "/Volumes/UVABX-PK/UVABX-PK-temp/BIOINFORMATICS-CORE/LarnerLab_Tom_M617TG5561LarnerRNAS16p_RNASe
q_Nov_2024/LarnerLab_Tom_M617TG5561LarnerRNAS16p_RNASeq_Nov_2024_Gene_Count_Table_U2KO_vs_U2W_res.txt", sep="\t",
row.names = FALSE) # need this for the volcano plot, edit to add

# write files for significant DEG's, FDR is 5%
res_sig <- subset(res, padj < 0.05)
write.table(res_sig, "/Volumes/UVABX-PK/UVABX-PK-temp/BIOINFORMATICS-CORE/LarnerLab_Tom_M617TG5561LarnerRNAS16p_R
NASeq_Nov_2024/LarnerLab_Tom_M617TG5561LarnerRNAS16p_RNASeq_Nov_2024_Gene_Count_Table_U2KO_vs_U2W_res_sig05.txt",
sep="\t",row.names = FALSE)
```

Volcano Plot: Contrast U2KO vs U2W (Group_Id_U2KO_vs_U2W)

Hide

```
################################ Volcano plot for Contrast U2KO vs U2W (Group_Id_U2KO_vs_U2W) ##################
######################

library(dplyr)
#library(calibrate)
library(ggrepel)
res <- read.table("/Volumes/UVABX-PK/UVABX-PK-temp/BIOINFORMATICS-CORE/LarnerLab_Tom_M617TG5561LarnerRNAS16p_RNAS
eq_Nov_2024/LarnerLab_Tom_M617TG5561LarnerRNAS16p_RNASeq_Nov_2024_Gene_Count_Table_U2KO_vs_U2W_res.txt", header=T
RUE)
#check for setting X limits
range(res$log2FoldChange) #
```

```
[1] -11.99804  10.53180
```

Hide

```
results = mutate(res, sig=ifelse(res$padj<0.05, "q-value < 0.05", "Not Significant"))
fc <- 1
results$diffexp <- "NO"
results$diffexp[results$log2FoldChange > fc & results$padj < 0.05] <- "UP"
results$diffexp[results$log2FoldChange < -fc & results$padj < 0.05] <- "DOWN"

#This line added later
results <- results %>% dplyr::filter ((!results$sig == "NA"))
#For Y axis lab
Y_axi_lab <- expression(bold(paste("-Log"[10] , " adjusted ", italic("p-value"))))
X_axi_lab <- expression(bold(paste("-Log" [2] , " fold change ")))
#This print grey and black (start)
p = ggplot(results, aes(log2FoldChange, -log10(padj), col = diffexp)) + ggtitle("Group_Id_U2KO_vs_U2W") +
  theme(plot.title = element_text(hjust = 0.5)) +
  geom_point(size=1) +
  scale_color_manual(values = c("#00AFBB", "grey", "#C71F3C"), labels = c("Downregulated", "Not significant", "Up
regulated")) +
  geom_vline(xintercept = c(-fc, fc), col = "gray", linetype = 'dashed') +
  geom_hline(yintercept = -log10(0.05), col = "gray", linetype = 'dashed') +
 xlim(-11, +10) + ylim(0, 100)
p = p+geom_text_repel(size = 3, data=dplyr::filter(results, padj<0.05,log2FoldChange>=fc), aes(label=row), max.ov
erlaps = 20)
p = p+geom_text_repel(size = 3, data=dplyr::filter(results, padj<0.05,log2FoldChange<=-fc), aes(label=row), max.o
verlaps = 20) + theme_bw() + theme(panel.border = element_blank(), panel.grid.major = element_blank(),
panel.grid.minor = element_blank(), axis.line = element_line(colour = "black")) +  theme(legend.text = element_te
xt(color = "black", size = 14, face="bold")) + theme(legend.title=element_blank())  + labs(y=Y_axi_lab, x=X_axi_l
ab) + theme(legend.position="bottom") + theme( axis.title.x = element_text(color="black", size=14, face="bold"),
axis.title.y = element_text(color="black", size=14, face="bold")) + theme(text = element_text(size=12, face="bol
d"))
p
```

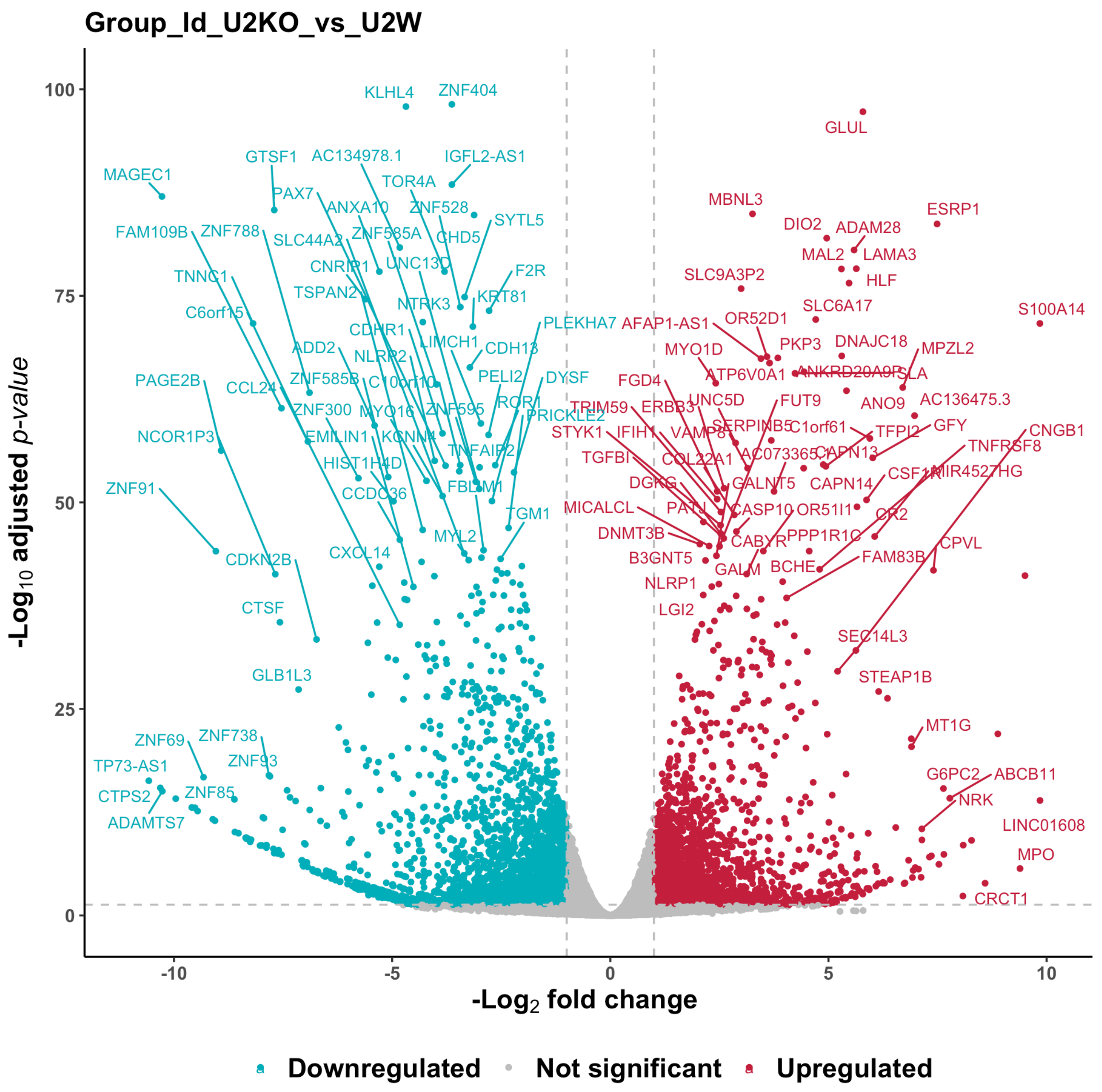

The up-regulated genes (log2FoldChange>=1 && padj<0.05) are in red text

The down-regulated genes (log2FoldChange<=-1 && padj<0.05) are in blue text

Heatmap of top 100 genes selected based on adjusted p -value

Contrast U2KO vs U2W (Group_Id_U2KO_vs_U2W)

Heatmap with U2KO and U2Wfrom this contrast

Hide

```
library("pheatmap")
#{r, warning=FALSE, echo=TRUE, include=TRUE, fig.width = 3, fig.height = 4}
#mat = assay(rld)[ head(order(res$padj),100), ] # select the top 100 genes with the lowest padj
mat=assay(rld)
#mat<-mat[,c(tmp_A,tmp_C)]
mat=subset(mat,rownames(mat) %in% res[head(order(res$padj),100),1]) #select the top 100 genes with the lowest pad
j
mat = mat - rowMeans(mat) # Subtract the row means from each value
colnames(mat)
```

```
[1] "u2k2_S6" "u2k3_S7" "u2k4_S8" "u2w1_S1" "u2w3_S3" "u2w4_S4"
```

Hide

```
#metadata1<-metadata[order(metadata$condition),]
df<-metadata
df = subset(df, select = -c(SampleName)) # Remove columns from annotation which are not required
#rownames(df) = colnames(mat)
rownames(df) = colnames(mat) # add rownames
# and plot the actual heatmap
pheatmap(mat, annotation_col=df, scale = "row", fontsize_row = 6.0,cluster_cols = TRUE)
```

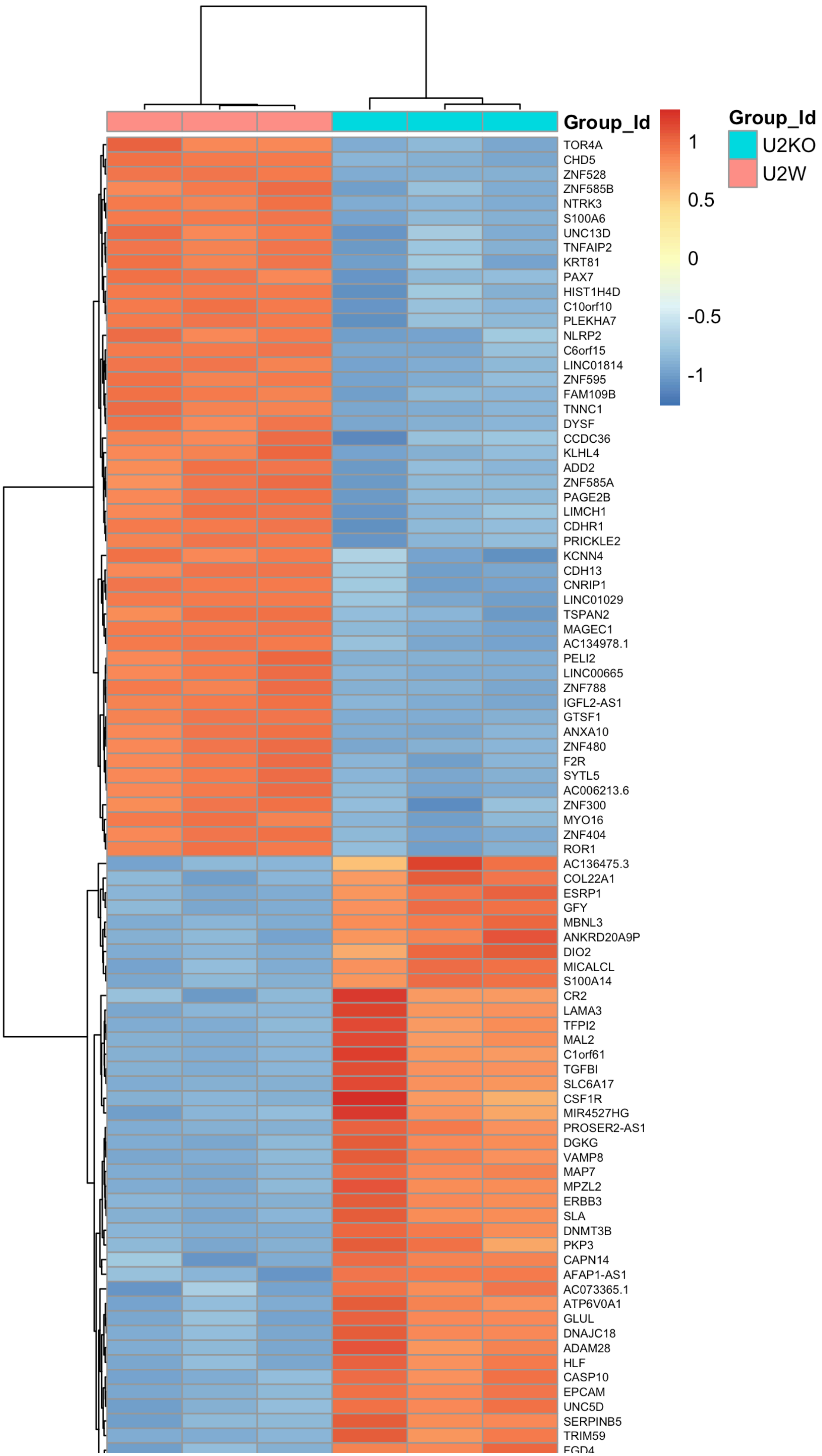

Group_Id
U2KO
U2W
1
0.5
0
-0.5
-1
TOR4A
CHD5
ZNF528
ZNF585B
NTRK3
S100A6
UNC13D
TNFAIP2
KRT81
PAX7
HIST1H4D
C10orf10
PLEKHA7
NLRP2
C6orf15
LINC01814
ZNF595
FAM109B
TNNC1
DYSF
CCDC36
KLHL4
ADD2
ZNF585A
PAGE2B
LIMCH1
CDHR1
PRICKLE2
KCNN4
CDH13
CNRIP1
LINC01029
TSPAN2
MAGEC1
AC134978.1
PELI2
LINC00665
ZNF788
IGFL2-AS1
GTSF1
ANXA10
ZNF480
F2R
SYTL5
AC006213.6
ZNF300
MYO16
ZNF404
ROR1
AC136475.3
COL22A1
ESRP1
GFY
MBNL3
ANKRD20A9P
DIO2
MICALCL
S100A14
CR2
LAMA3
TFPI2
MAL2
C1orf61
TGFBI
SLC6A17
CSF1R
MIR4527HG
PROSER2-AS1
DGKG
VAMP8
MAP7
MPZL2
ERBB3
SLA
DNMT3B
PKP3
CAPN14
AFAP1-AS1
AC073365.1
ATP6V0A1
GLUL
DNAJC18
ADAM28
HLF
CASP10
EPCAM
UNC5D
SERPINB5
TRIM59
FGD4

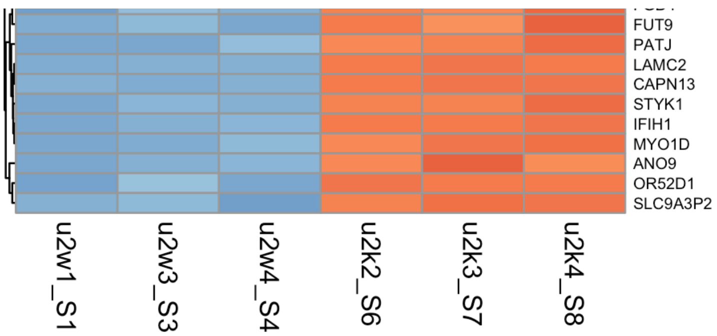

Loading provided gene lists

genelist1

Hide

```
genelist<-read.csv2(file="~/Desktop/BIOINFORMATICS-CORE/LarnerLab_Tom_M617TG5561LarnerRNAS16p_RNASeq_Nov_2024/gen
elist1_ALT_TERT_pathway.txt",sep="\t",header=FALSE)
```

Hide

```
table(genelist$V2)
```

```

Alternative Lengthening Telomere                      TERT pathway 
                              99                                24 
```

Hide

```
ALT_pathway_genelist<-genelist[genelist$V2=="Alternative Lengthening Telomere ",1]

ALT_pathway_genelist_unique<-ALT_pathway_genelist[!duplicated(ALT_pathway_genelist)]

TERT_pathway_genelist<-genelist[genelist$V2=="TERT pathway ",1]
```

Heatmap of unique gene list related to Alternative Lengthening Telomere pathway

Contrast U2KO vs U2W (Group_Id_U2KO_vs_U2W)

Hide

```
library("pheatmap")
#mat = assay(rld)[ head(order(res$padj),100), ] # select the top 100 genes with the lowest padj
# select specific samples
mat=dplyr::select(as.data.frame(assay(rld)),c("u2k2_S6","u2k3_S7","u2k4_S8","u2w1_S1","u2w3_S3","u2w4_S4"))
mat <- as.matrix(filter(mat, rownames(mat) %in% ALT_pathway_genelist_unique))
mat = mat - rowMeans(mat) # Subtract the row means from each value
# Optional, but to make the plot nicer:
df = as.data.frame(colData(rld)[,c("Group_Id")]) # Create a dataframe with a column of the conditions
colnames(df) = "group" # Rename the column header
#df <- filter(df, df$group %in% c("Control","DHT_24h"))
rownames(df) = colnames(mat) # add rownames
# and plot the actual heatmap
pheatmap(mat, annotation_col=df, scale = "row", fontsize_row = 6.0, cluster_cols = F)
```

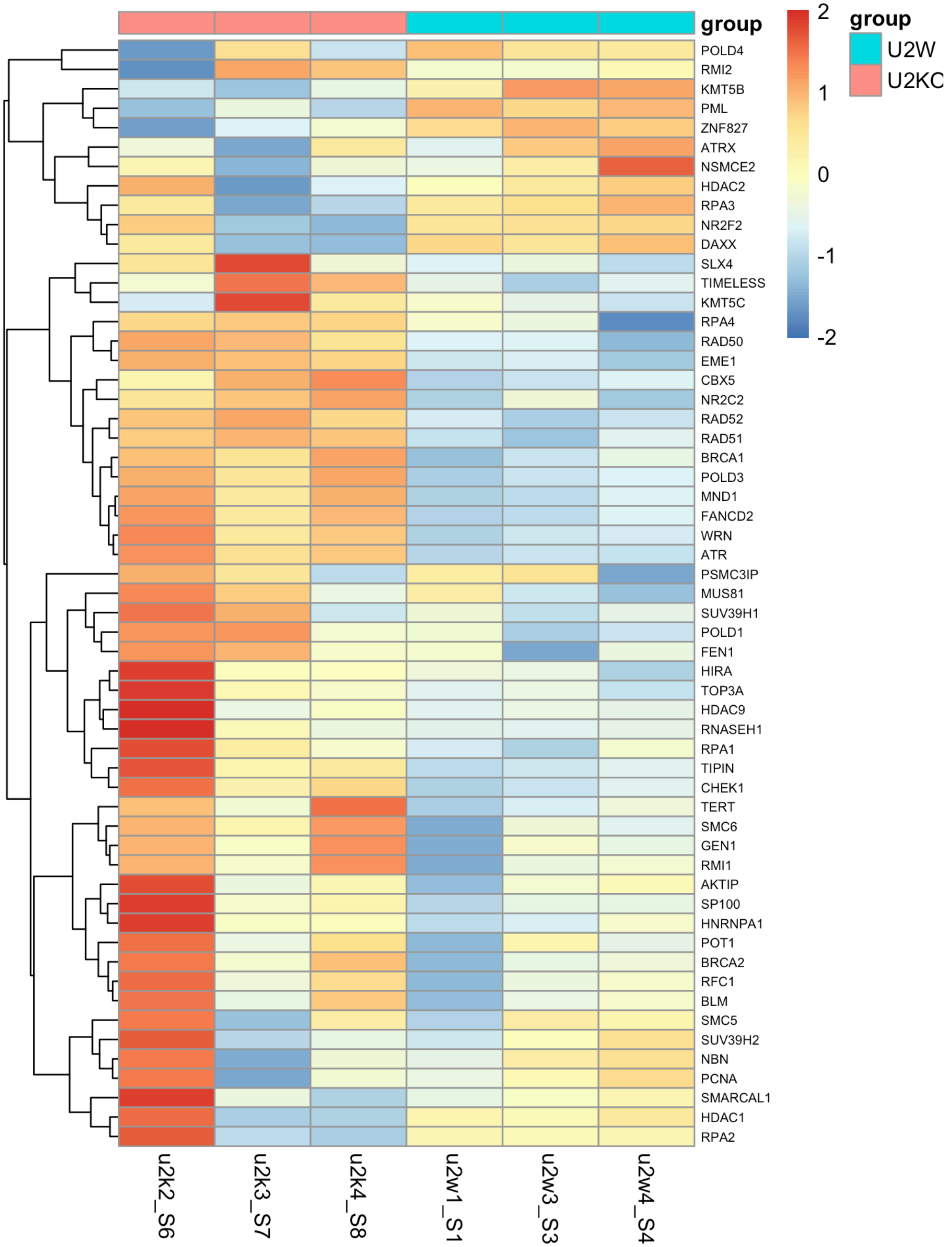

Heatmap of unique gene list related to Alternative Lengthening Telomere pathway

All 16 samples

Hide

```
library("pheatmap")
mat<-as.data.frame(assay(rld_16))
mat <- as.matrix(filter(mat, rownames(mat) %in% ALT_pathway_genelist_unique))
mat = mat - rowMeans(mat) # Subtract the row means from each value
# Optional, but to make the plot nicer:
df = as.data.frame(colData(rld_16)[,c("Group_Id")]) # Create a dataframe with a column of the conditions
colnames(df) = "group" # Rename the column header
#df <- filter(df, df$group %in% c("Control","DHT_24h"))
rownames(df) = colnames(mat) # add rownames
# and plot the actual heatmap
pheatmap(mat, annotation_col=df, scale = "row", fontsize_row = 6.0, cluster_cols = FALSE)
```

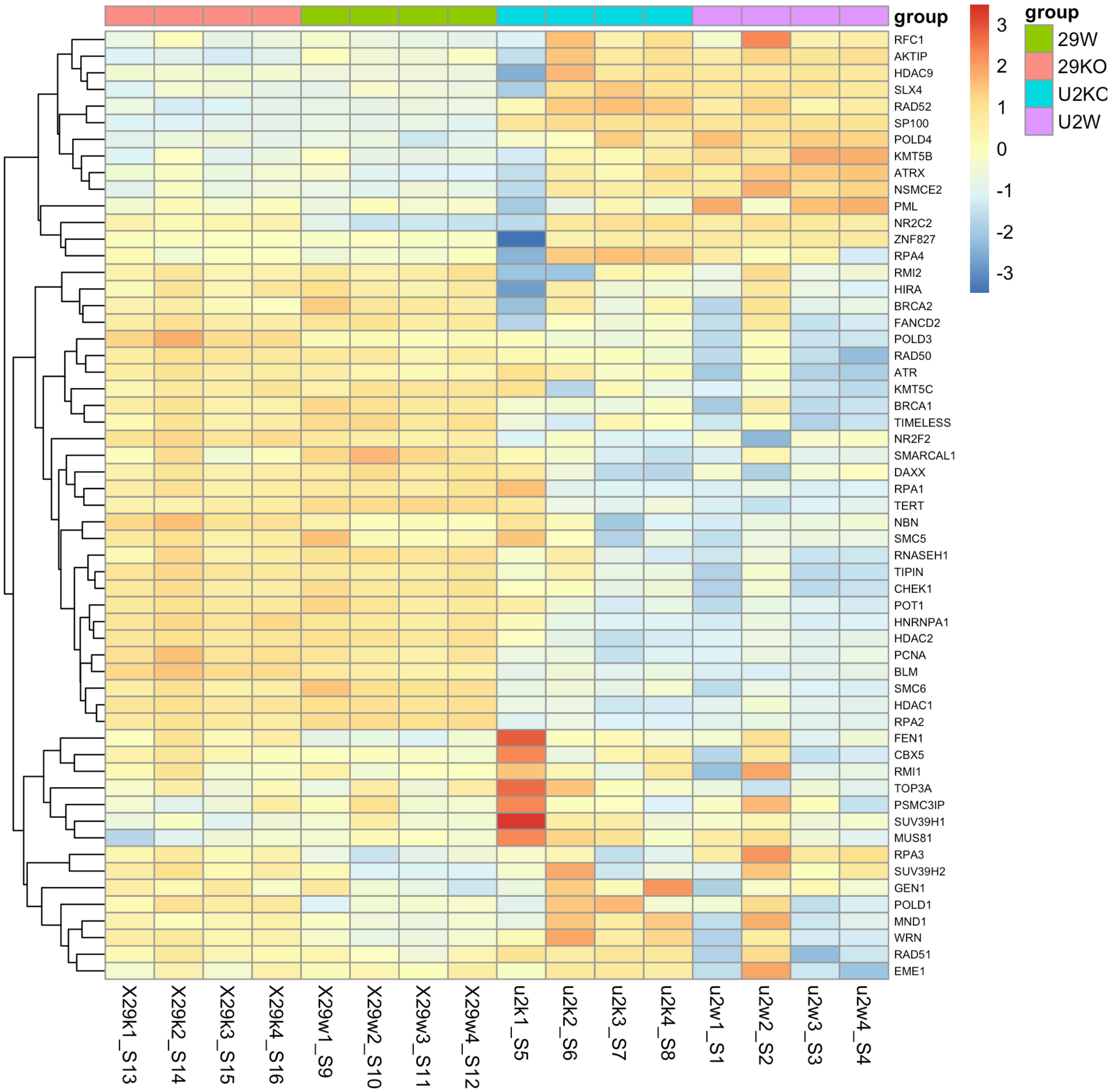

Heatmap of unique gene list related to TERT pathway

Contrast U2KO vs U2W (Group_Id_U2KO_vs_U2W)

Hide

```
library("pheatmap")
#mat = assay(rld)[ head(order(res$padj),100), ] # select the top 100 genes with the lowest padj
# select specific samples
mat=dplyr::select(as.data.frame(assay(rld)),c("u2k2_S6","u2k3_S7","u2k4_S8","u2w1_S1","u2w3_S3","u2w4_S4"))
mat <- as.matrix(filter(mat, rownames(mat) %in% TERT_pathway_genelist))
mat = mat - rowMeans(mat) # Subtract the row means from each value
# Optional, but to make the plot nicer:
df = as.data.frame(colData(rld)[,c("Group_Id")]) # Create a dataframe with a column of the conditions
colnames(df) = "group" # Rename the column header
#df <- filter(df, df$group %in% c("Control","DHT_24h"))
rownames(df) = colnames(mat) # add rownames
# and plot the actual heatmap
pheatmap(mat, annotation_col=df, scale = "row", fontsize_row = 6.0, cluster_cols = F)
```

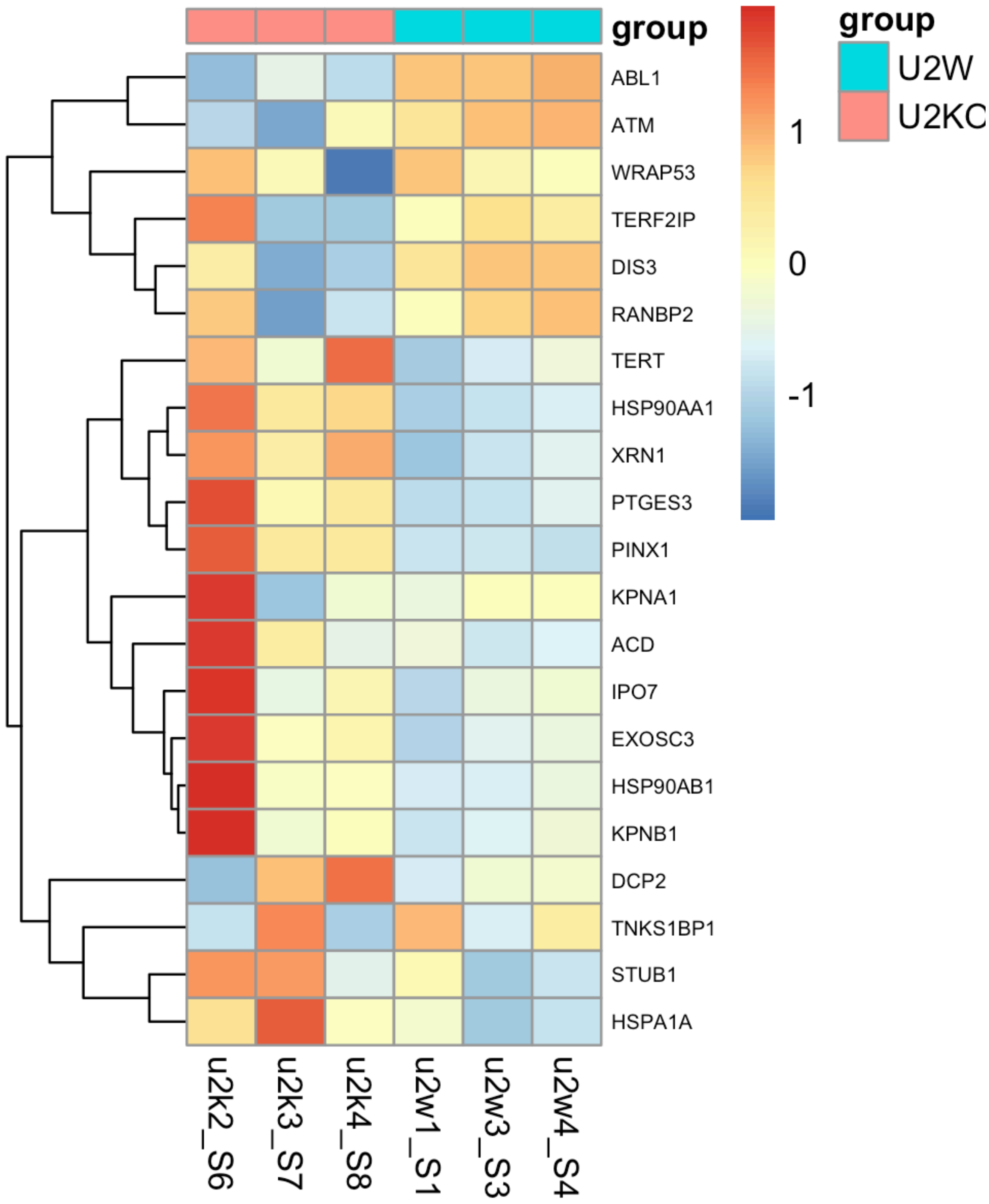

Heatmap of unique gene list related to TERT pathway

all 16 samples

Hide

```
library("pheatmap")
mat<-as.data.frame(assay(rld_16))
mat <- as.matrix(filter(mat, rownames(mat) %in% TERT_pathway_genelist))
mat = mat - rowMeans(mat) # Subtract the row means from each value
# Optional, but to make the plot nicer:
df = as.data.frame(colData(rld_16)[,c("Group_Id")]) # Create a dataframe with a column of the conditions
colnames(df) = "group" # Rename the column header
#df <- filter(df, df$group %in% c("Control","DHT_24h"))
rownames(df) = colnames(mat) # add rownames
# and plot the actual heatmap
pheatmap(mat, annotation_col=df, scale = "row", fontsize_row = 6.0, cluster_cols = FALSE)
```

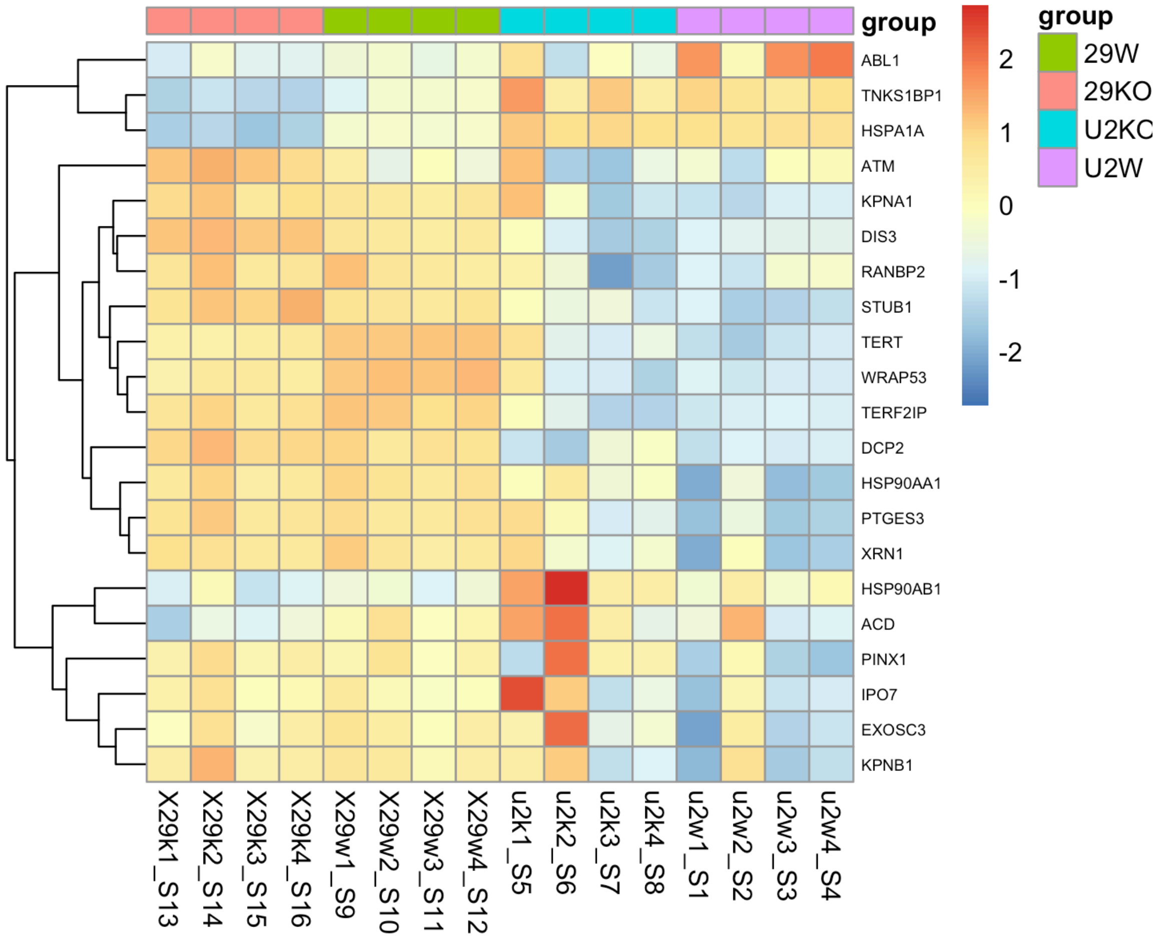

combined pathway genes list

Hide

```
combined_pathway_genelist<-c(ALT_pathway_genelist_unique,TERT_pathway_genelist)
```

Heatmap of combined pathway genelist

Contrast U2KO vs U2W (Group_Id_U2KO_vs_U2W)

Hide

```
library("pheatmap")
#mat = assay(rld)[ head(order(res$padj),100), ] # select the top 100 genes with the lowest padj
# select specific samples
mat=dplyr::select(as.data.frame(assay(rld)),c("u2k2_S6","u2k3_S7","u2k4_S8","u2w1_S1","u2w3_S3","u2w4_S4"))
mat <- as.matrix(filter(mat, rownames(mat) %in% combined_pathway_genelist))
mat = mat - rowMeans(mat) # Subtract the row means from each value
# Optional, but to make the plot nicer:
df = as.data.frame(colData(rld)[,c("Group_Id")]) # Create a dataframe with a column of the conditions
colnames(df) = "group" # Rename the column header
#df <- filter(df, df$group %in% c("Control","DHT_24h"))
rownames(df) = colnames(mat) # add rownames
# and plot the actual heatmap
pheatmap(mat, annotation_col=df, scale = "row", fontsize_row = 6.0, cluster_cols = F)
```

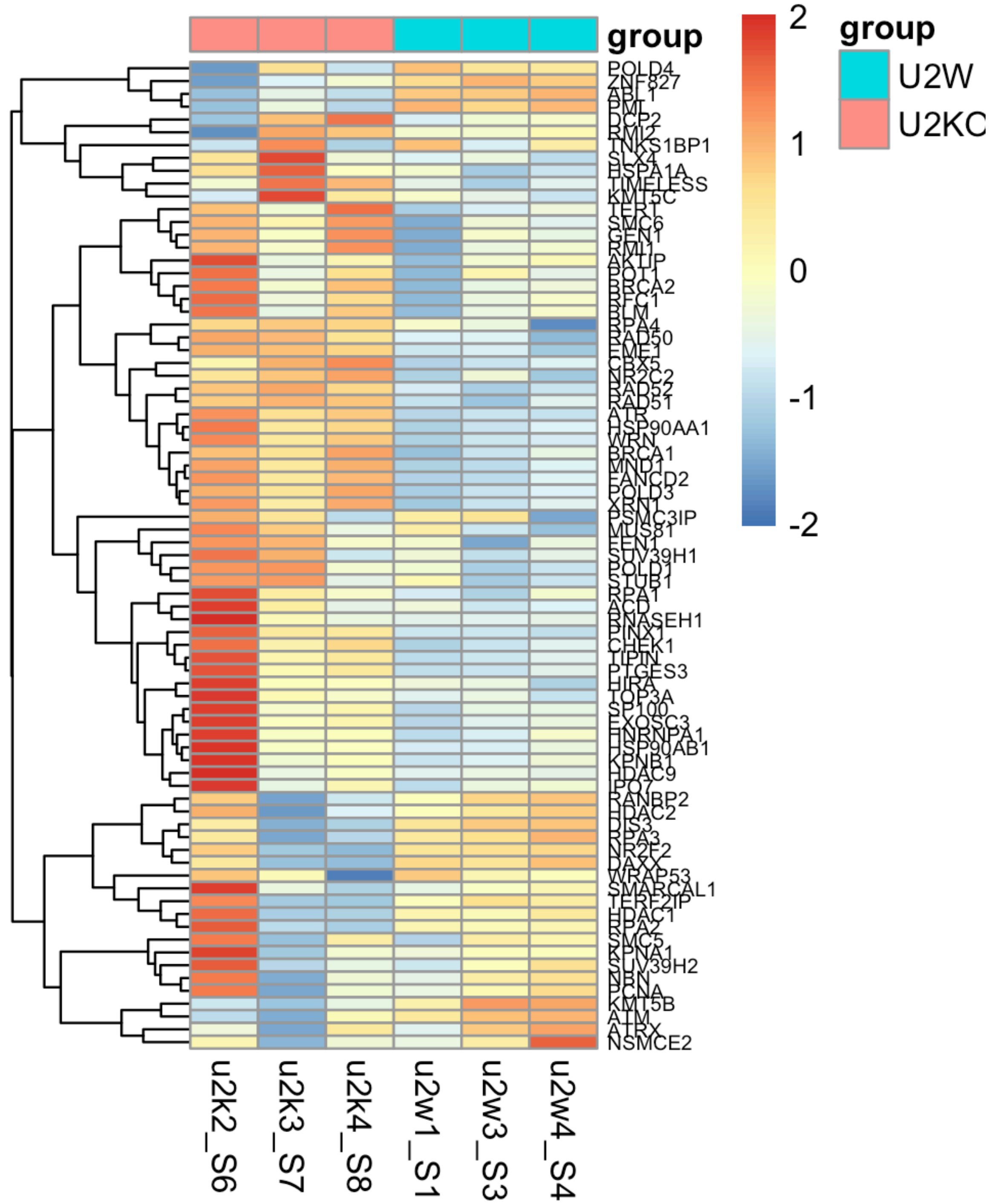

Heatmap of combined pathway genelist

all 16 samples

Hide

```
library("pheatmap")
mat<-as.data.frame(assay(rld_16))
mat <- as.matrix(filter(mat, rownames(mat) %in% combined_pathway_genelist))
mat = mat - rowMeans(mat) # Subtract the row means from each value
# Optional, but to make the plot nicer:
df = as.data.frame(colData(rld_16)[,c("Group_Id")]) # Create a dataframe with a column of the conditions
colnames(df) = "group" # Rename the column header
#df <- filter(df, df$group %in% c("Control","DHT_24h"))
rownames(df) = colnames(mat) # add rownames
# and plot the actual heatmap
pheatmap(mat, annotation_col=df, scale = "row", fontsize_row = 6.0, cluster_cols = FALSE)
```

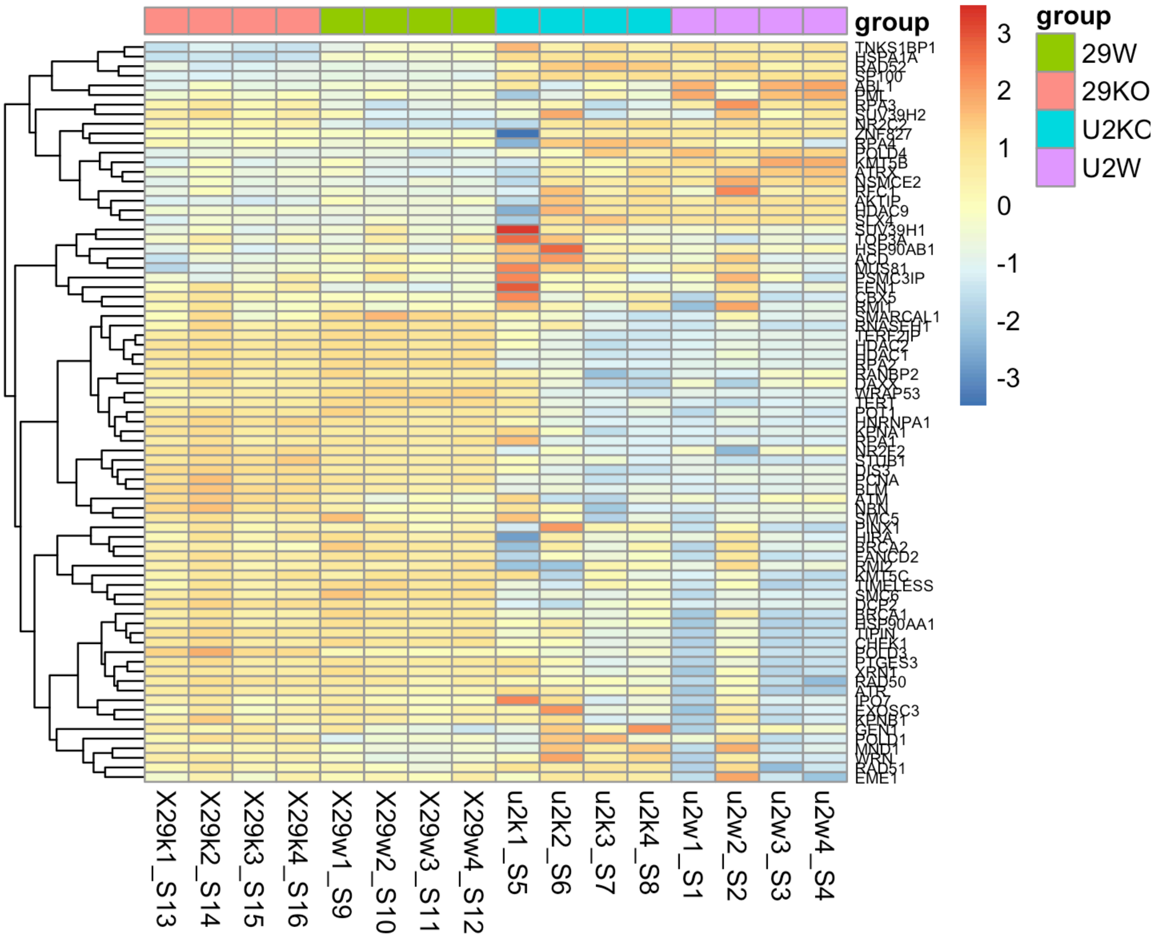

Pathway Analysis Contrast U2KO vs U2W (Group_Id_U2KO_vs_U2W)

Use the DESeq result. Here all you'll care is the gene symbol and the test statistic. Get just those columns from res variable.

Hide

```
res2 <- res %>%
  dplyr::select(row, stat) %>%
  na.omit() %>%
  distinct() %>%
  group_by (row) %>%
  summarize(stat=mean(stat))
```

Now create named vector of test statistics.

Hide

```
ranks <- deframe(res2)
head(ranks, 20)
```

```
     A1BG   A1BG-AS1       A1CF        A2M    A2M-AS1      A2ML1     A4GALT       AAAS       AACS     AACSP1 
0.1656653 -0.0853183 -3.1911045 -8.4806214 -7.2039222  0.4824952 -1.8603315 -0.3106115 -1.6174032 -6.1809184 
    AADAC    AADACL3   AADACP1      AADAT      AAED1      AAGAB       AAK1      AAMDC       AAMP      AANAT 
0.6590152  2.7023820  0.8275953  3.3755685  1.5149084  0.6954215 -2.9351528 -2.7392887  0.5814206  6.9485250 
```

Pathway analysis using clusterprofiler

Pathway dataset : MSigDB hallmark dataset

#GSEA function below requires sorted gene rank list

Hide

```
#ranks[1:10]

ranks_sorted<-sort(ranks,decreasing = TRUE)

ranks_sorted[1:10]
```

```
     LAMC2      EPCAM       MAP7 PROSER2-AS1       GLUL      MBNL3      ESRP1       DIO2     ADAM28
  28.40321   23.02080   22.04606   21.70331   21.38516   19.99796   19.85053   19.64817   19.47428
     LAMA3
  19.20294
```

GSEA based on MSigDB hallmark dataset

H represents Hallmark dataset

Hide

```
msigdb<- msigdbr(species = "Homo sapiens", category = "H") %>% dplyr::select(gs_name, gene_symbol)
```

Hide

```
ewp <- GSEA(ranks_sorted, TERM2GENE=msigdb)
```

```
using 'fgsea' for GSEA analysis, please cite Korotkevich et al (2019).

preparing geneSet collections...
GSEA analysis...
Warning in preparePathwaysAndStats(pathways, stats, minSize, maxSize, gseaParam,  :
  There are ties in the preranked stats (0.06% of the list).
The order of those tied genes will be arbitrary, which may produce unexpected results.
Warning in fgseaMultilevel(pathways = pathways, stats = stats, minSize = minSize,  :
  For some pathways, in reality P-values are less than 1e-10. You can set the `eps` argument to zero for better e
stimation.
leading edge analysis...
done...
```

Hide

```
head (ewp@result)
```

	ID <chr>	Description <chr>	setSize <int>	enrichmentScore <dbl>
HALLMARK_MYC_TARGETS_V2	HALLMARK_MYC_TARGETS_V2	HALLMARK_MYC_TARGETS_V2	58	0.7471268
HALLMARK_MYC_TARGETS_V1	HALLMARK_MYC_TARGETS_V1	HALLMARK_MYC_TARGETS_V1	196	0.5508426
HALLMARK_E2F_TARGETS	HALLMARK_E2F_TARGETS	HALLMARK_E2F_TARGETS	197	0.4668031
HALLMARK_P53_PATHWAY	HALLMARK_P53_PATHWAY	HALLMARK_P53_PATHWAY	193	-0.4524655
HALLMARK_MYOGENESIS	HALLMARK_MYOGENESIS	HALLMARK_MYOGENESIS	186	-0.4226629
HALLMARK_COAGULATION	HALLMARK_COAGULATION	HALLMARK_COAGULATION	113	-0.4336207

6 rows | 1-5 of 11 columns

Hide

```
fgseaResTidy <- ewp %>%
  as_tibble() %>%
  arrange(desc(NES))


fgseaResTidy %>%
  dplyr::select(-Description) %>%
  arrange(p.adjust) %>%
  DT::datatable()
```

Show 10 entries

Search:

	ID	setSize	enrichmentScore	NES	pvalue
1	HALLMARK_MYC_TARGETS_V2	58	0.7471267609405172	2.671753353032893	1e-10

	ID	setSize	enrichmentScore	NES	pvalue	
2	HALLMARK_MYC_TARGETS_V1	196	0.5508426441921561	2.362196459378316	1e-10	
3	HALLMARK_E2F_TARGETS	197	0.4668031105958554	2.009107546568051	1.327265266190991e-8	
4	HALLMARK_P53_PATHWAY	193	-0.4524655175008447	-1.837386414237382	0.000001046045587633861	0.0
5	HALLMARK_MYOGENESIS	186	-0.4226628751137489	-1.710723310859783	0.00005238141362151463	0.
6	HALLMARK_EPITHELIAL_MESENCHYMAL_TRANSITION	193	-0.3839847704562998	-1.559297611025458	0.0007477215823832679	[illegible]
7	HALLMARK_UV_RESPONSE_DN	140	-0.4184419689835827	-1.645214562699757	0.0009214534375418305	[illegible]
8	HALLMARK_COAGULATION	113	-0.4336207128513209	-1.656110822245195	0.0008098759846424784	[illegible]
9	HALLMARK_HEDGEHOG_SIGNALING	33	-0.6096578528074283	-1.850004749567896	0.001242975127819723	[illegible]
10	HALLMARK_APOPTOSIS	151	-0.3928588102225308	-1.560387469521027	0.001953411500549312	[illegible]

Showing 1 to 10 of 16 entries

Previous 1 2 Next

Hide

```
library(dplyr)
```

Arranging pathway enrichment list based on NES values

Hide

```
ewp2 <- arrange(ewp, desc(abs(NES))) %>% group_by(sign(NES))
```

Hide

```
ggplot(ewp2, showCategory=20,aes(NES, fct_reorder(Description, NES),fill=qvalue)) + geom_col() +
scale_fill_gradientn(colours=c("#b3eebe", "#46bac2", "#371ea3"),
guide=guide_colorbar(reverse=TRUE))+
xlab("Normalized Enrichment Score") + ylab(NULL) +
  ggtitle("Hallmark pathways")
```

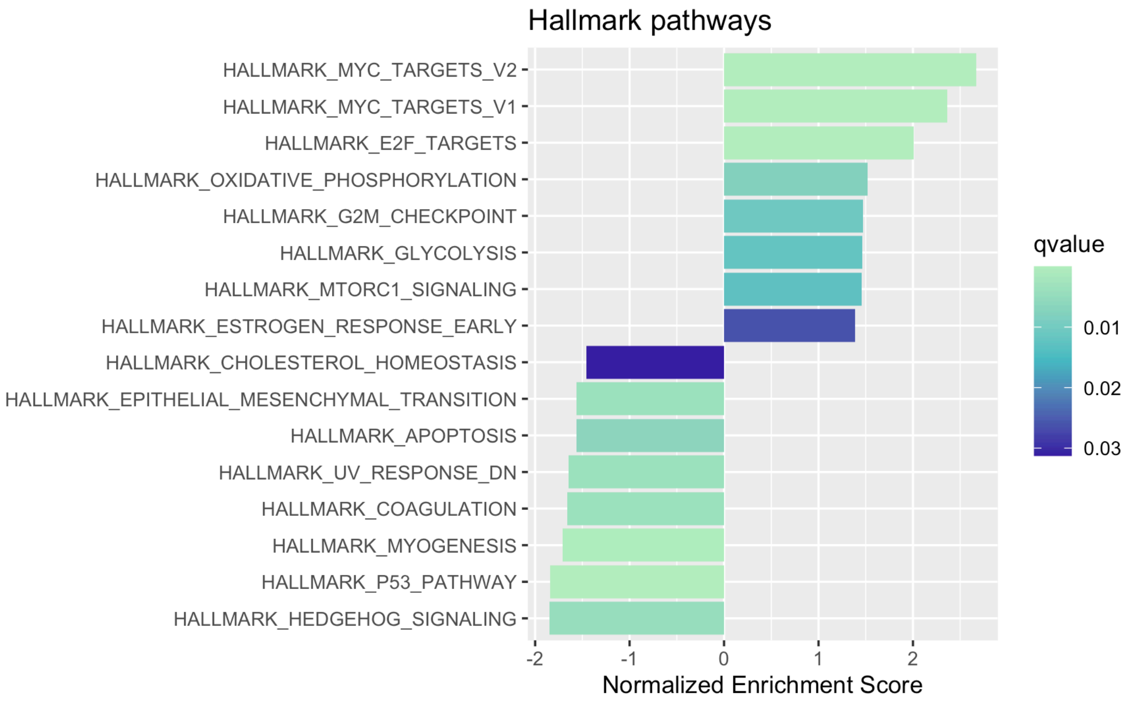

Hide

```
ewp3 <- arrange(ewp, desc(abs(NES)))
```

visualisation using clusterprofiler

Hide

```
## visualization
color <- c("#f7ca64", "#43a5bf", "#86c697", "#a670d6", "#ef998a","#f7ca64", "#43a5bf", "#86c697", "#a670d6", "#ef
998a")
ech4 <- gseaplot2(ewp3, 1:10, color = color, pvalue_table=F, base_size=10)

ech4
```

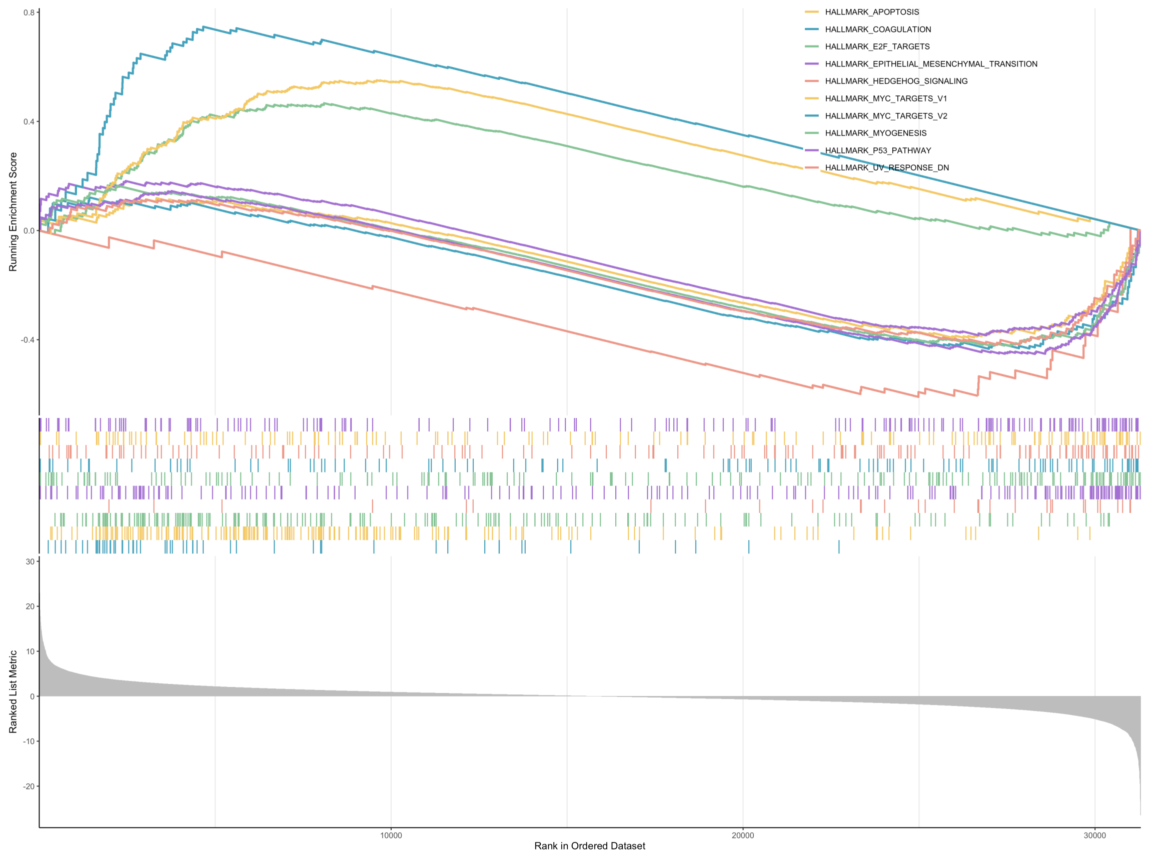

Hide

```
ech3 <- upsetplot(ewp3, n=10)

ech3
```

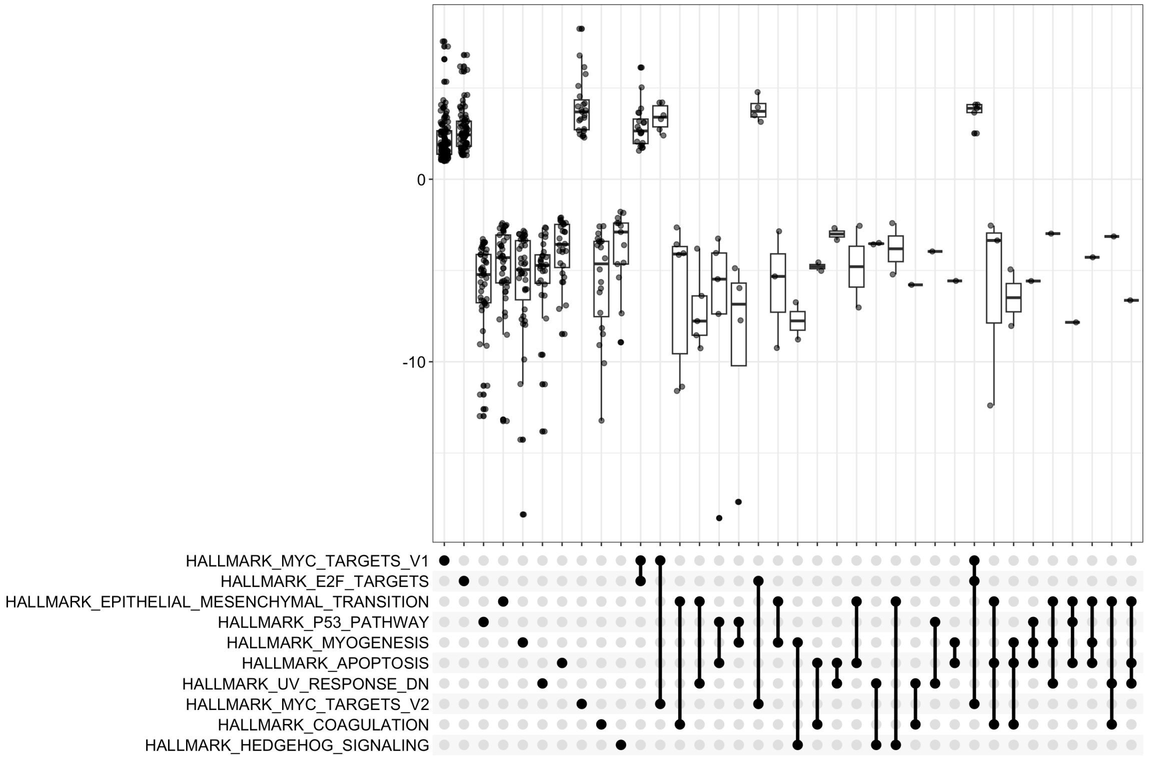

HALLMARK_DNA_REPAIR gene set

Hide

```
pathway_genes<-msigdb[msigdb$gs_name=="HALLMARK_DNA_REPAIR",]$gene_symbol
```

Hide

```
pathway_genes
```

```
  [1] "AAAS"    "ADA"     "ADCY6"   "ADRM1"   "AGO4"    "AK1"     "AK3"     "ALYREF"  "APRT"    "ARL6IP1"
 [11] "BCAM"    "BCAP31"  "BOLA2"   "BRF2"    "CANT1"   "CCNO"    "CDA"     "CETN2"   "CLP1"    "CMPK2"
 [21] "COX17"   "CSTF3"   "DAD1"    "DCTN4"   "DDB1"    "DDB2"    "DGCR8"   "DGUOK"   "DUT"     "EDF1"
 [31] "EIF1B"   "ELL"     "ELOA"    "ERCC1"   "ERCC2"   "ERCC3"   "ERCC4"   "ERCC5"   "ERCC8"   "FEN1"
 [41] "GMPR2"   "GMPR2"   "GPX4"    "GSDME"   "GTF2A2"  "GTF2B"   "GTF2F1"  "GTF2H1"  "GTF2H1"  "GTF2H3"
 [51] "GTF2H5"  "GTF3C5"  "GUK1"    "HCLS1"   "HPRT1"   "IMPDH2"  "ITPA"    "LIG1"    "MPC2"    "MPG"
 [61] "MRPL40"  "NCBP2"   "NELFB"   "NELFCD"  "NELFE"   "NELFE"   "NELFE"   "NELFE"   "NELFE"   "NELFE"
 [71] "NFX1"    "NME1"    "NME3"    "NME4"    "NPR2"    "NT5C"    "NT5C3A"  "NUDT21"  "NUDT9"   "PCNA"
 [81] "PDE4B"   "PDE6G"   "PNP"     "POLA1"   "POLA2"   "POLB"    "POLD1"   "POLD3"   "POLD4"   "POLE4"
 [91] "POLH"    "POLL"    "POLR1C"  "POLR1D"  "POLR1H"  "POLR1H"  "POLR1H"  "POLR1H"  "POLR1H"  "POLR1H"
[101] "POLR1H"  "POLR1H"  "POLR1H"  "POLR2A"  "POLR2A"  "POLR2C"  "POLR2D"  "POLR2E"  "POLR2F"  "POLR2G"
[111] "POLR2H"  "POLR2I"  "POLR2J"  "POLR2K"  "POLR3C"  "POLR3GL" "POM121"  "PRIM1"   "RAD51"   "RAD52"
[121] "RAE1"    "RALA"    "RBX1"    "REV3L"   "RFC2"    "RFC3"    "RFC4"    "RFC5"    "RNMT"    "RPA2"
[131] "RPA3"    "RRM2B"   "SAC3D1"  "SDCBP"   "SEC61A1" "SF3A3"   "SMAD5"   "SNAPC4"  "SNAPC5"  "SRSF6"
[141] "SSRP1"   "STX3"    "SUPT4H1" "SUPT5H"  "SURF1"   "SURF1"   "TAF10"   "TAF12"   "TAF13"   "TAF1C"
[151] "TAF6"    "TAF9"    "TAF9"    "TARBP2"  "TK2"     "TMED2"   "TP53"    "TSG101"  "TYMS"    "UMPS"
[161] "UPF3B"   "USP11"   "VPS28"   "VPS28"   "VPS37B"  "VPS37D"  "XPC"     "ZNF707"  "ZNF707"  "ZWINT"
```

Heatmap for genes in HALLMARK_DNA_REPAIR

All 16 samples

Hide

```
library("pheatmap")
mat<-as.data.frame(assay(rld_16))
mat <- as.matrix(filter(mat, rownames(mat) %in% pathway_genes))
mat = mat - rowMeans(mat) # Subtract the row means from each value
# Optional, but to make the plot nicer:
df = as.data.frame(colData(rld_16)[,c("Group_Id")]) # Create a dataframe with a column of the conditions
colnames(df) = "group" # Rename the column header
#df <- filter(df, df$group %in% c("Control","DHT_24h"))
rownames(df) = colnames(mat) # add rownames
# and plot the actual heatmap
pheatmap(mat, annotation_col=df, scale = "row", fontsize_row = 6.0, cluster_cols = FALSE)
```

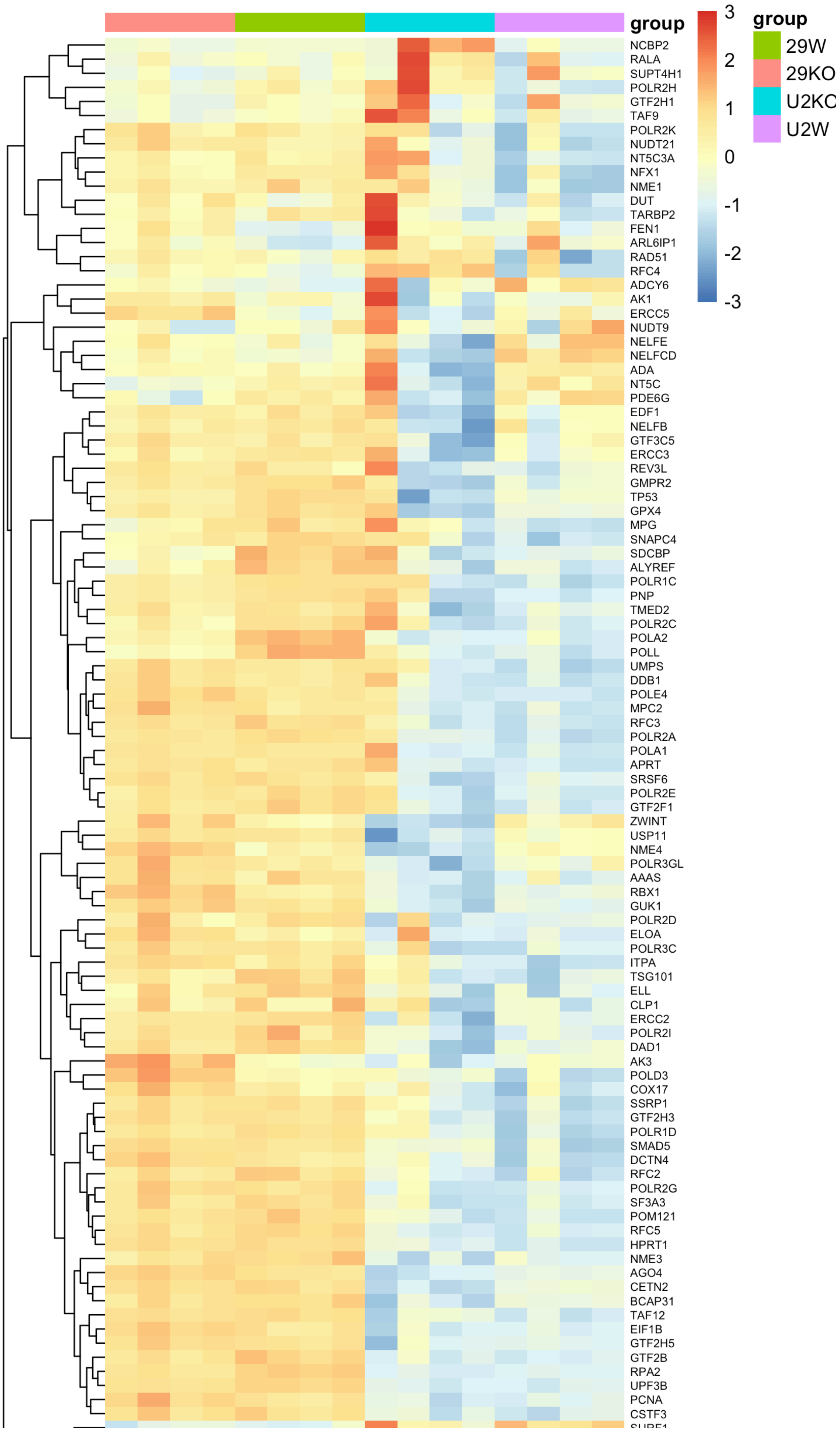
group
3
2
1
0
-1
-2
-3
group
29W
29KO
U2KC
U2W
NCBP2
RALA
SUPT4H1
POLR2H
GTF2H1
TAF9
POLR2K
NUDT21
NT5C3A
NFX1
NME1
DUT
TARBP2
FEN1
ARL6IP1
RAD51
RFC4
ADCY6
AK1
ERCC5
NUDT9
NELFE
NELFCD
ADA
NT5C
PDE6G
EDF1
NELFB
GTF3C5
ERCC3
REV3L
GMPR2
TP53
GPX4
MPG
SNAPC4
SDCBP
ALYREF
POLR1C
PNP
TMED2
POLR2C
POLA2
POLL
UMPS
DDB1
POLE4
MPC2
RFC3
POLR2A
POLA1
APRT
SRSF6
POLR2E
GTF2F1
ZWINT
USP11
NME4
POLR3GL
AAAS
RBX1
GUK1
POLR2D
ELOA
POLR3C
ITPA
TSG101
ELL
CLP1
ERCC2
POLR2I
DAD1
AK3
POLD3
COX17
SSRP1
GTF2H3
POLR1D
SMAD5
DCTN4
RFC2
POLR2G
SF3A3
POM121
RFC5
HPRT1
NME3
AGO4
CETN2
BCAP31
TAF12
EIF1B
GTF2H5
GTF2B
RPA2
UPF3B
PCNA
CSTF3

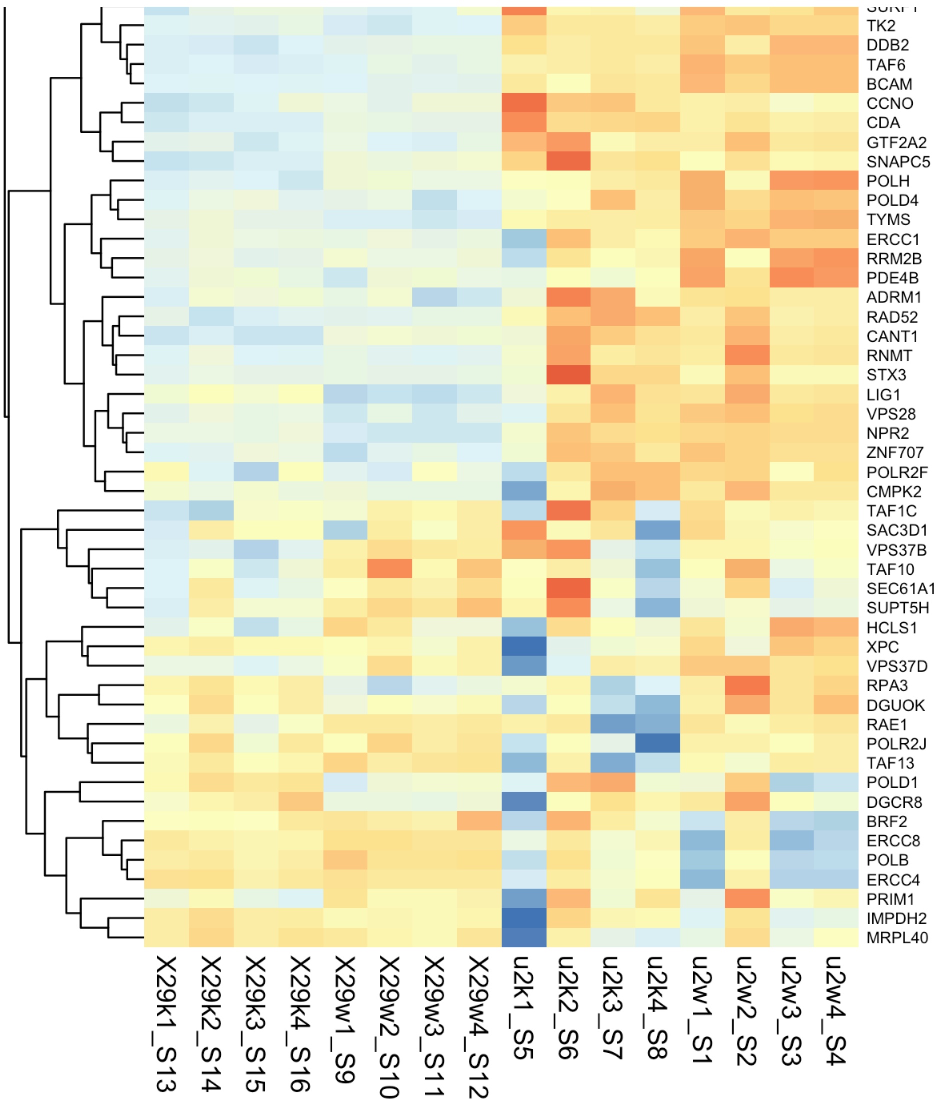

HALLMARK_EPITHELIAL_MESENCHYMAL_TRANSITION

Hide

```
pathway_genes<-msigdb[msigdb$gs_name=="HALLMARK_EPITHELIAL_MESENCHYMAL_TRANSITION",]$gene_symbol
```

Hide

```
pathway_genes
```

```
  [1] "ABI3BP"     "ACTA2"      "ADAM12"     "ANPEP"      "APLP1"      "AREG"       "BASP1"      "BDNF"       "BGN"
 [10] "BMP1"       "CADM1"      "CALD1"      "CALU"       "CAP2"       "CAPG"       "CCN1"       "CCN2"       "CD44"
 [19] "CD59"       "CDH11"      "CDH2"       "CDH6"       "COL11A1"    "COL12A1"    "COL16A1"    "COL1A1"     "COL1A2"
 [28] "COL3A1"     "COL4A1"     "COL4A2"     "COL5A1"     "COL5A2"     "COL5A3"     "COL6A2"     "COL6A3"     "COL7A1"
 [37] "COL8A2"     "COLGALT1"   "COMP"       "COPA"       "CRLF1"      "CTHRC1"     "CXCL1"      "CXCL12"     "CXCL6"
 [46] "CXCL8"      "DAB2"       "DCN"        "DKK1"       "DPYSL3"     "DST"        "ECM1"       "ECM2"       "EDIL3"
 [55] "EFEMP2"     "ELN"        "EMP3"       "ENO2"       "FAP"        "FAS"        "FBLN1"      "FBLN2"      "FBLN5"
 [64] "FBN1"       "FBN2"       "FERMT2"     "FGF2"       "FLNA"       "FMOD"       "FN1"        "FOXC2"      "FSTL1"
 [73] "FSTL3"      "FUCA1"      "FZD8"       "GADD45A"    "GADD45B"    "GAS1"       "GEM"        "GJA1"       "GLIPR1"
 [82] "GPC1"       "GPX7"       "GREM1"      "GREM1"      "GREM1"      "HTRA1"      "ID2"        "IGFBP2"     "IGFBP3"
 [91] "IGFBP4"     "IL15"       "IL32"       "IL6"        "INHBA"      "ITGA2"      "ITGA5"      "ITGAV"      "ITGB1"
[100] "ITGB3"      "ITGB5"      "JUN"        "LAMA1"      "LAMA2"      "LAMA3"      "LAMC1"      "LAMC2"      "LGALS1"
[109] "LOX"        "LOXL1"      "LOXL2"      "LRP1"       "LRRC15"     "LUM"        "MAGEE1"     "MATN2"      "MATN3"
[118] "MCM7"       "MEST"       "MFAP5"      "MGP"        "MMP1"       "MMP1"       "MMP14"      "MMP2"       "MMP3"
[127] "MSX1"       "MXRA5"      "MYL9"       "MYLK"       "NID2"       "NNMT"       "NOTCH2"     "NT5E"       "NTM"
[136] "OXTR"       "P3H1"       "PCOLCE"     "PCOLCE2"    "PDGFRB"     "PDLIM4"     "PFN2"       "PLAUR"      "PLOD1"
[145] "PLOD2"      "PLOD3"      "PMEPA1"     "PMP22"      "POSTN"      "PPIB"       "PRRX1"      "PRSS2"      "PRSS2"
[154] "PTHLH"      "PTX3"       "PVR"        "QSOX1"      "RGS4"       "RHOB"       "SAT1"       "SCG2"       "SDC1"
[163] "SDC4"       "SERPINE1"   "SERPINE2"   "SERPINH1"   "SFRP1"      "SFRP4"      "SGCB"       "SGCD"       "SGCG"
[172] "SLC6A8"     "SLIT2"      "SLIT3"      "SNAI2"      "SNTB1"      "SPARC"      "SPOCK1"     "SPP1"       "TAGLN"
[181] "TFPI2"      "TGFB1"      "TGFBI"      "TGFBR3"     "TGM2"       "THBS1"      "THBS2"      "THY1"       "TIMP1"
[190] "TIMP3"      "TNC"        "TNFAIP3"    "TNFRSF11B"  "TNFRSF12A"  "TPM1"       "TPM2"       "TPM4"       "VCAM1"
[199] "VCAN"       "VEGFA"      "VEGFC"      "VIM"        "WIPF1"      "WNT5A"
```

Heatmap for genes in HALLMARK_EPITHELIAL_MESENCHYMAL_TRANSITION

All 16 samples

Hide

```
library("pheatmap")
mat<-as.data.frame(assay(rld_16))
mat <- as.matrix(filter(mat, rownames(mat) %in% pathway_genes))
mat = mat - rowMeans(mat) # Subtract the row means from each value
# Optional, but to make the plot nicer:
df = as.data.frame(colData(rld_16)[,c("Group_Id")]) # Create a dataframe with a column of the conditions
colnames(df) = "group" # Rename the column header
#df <- filter(df, df$group %in% c("Control","DHT_24h"))
rownames(df) = colnames(mat) # add rownames
# and plot the actual heatmap
pheatmap(mat, annotation_col=df, scale = "row", fontsize_row = 6.0, cluster_cols = FALSE)
```

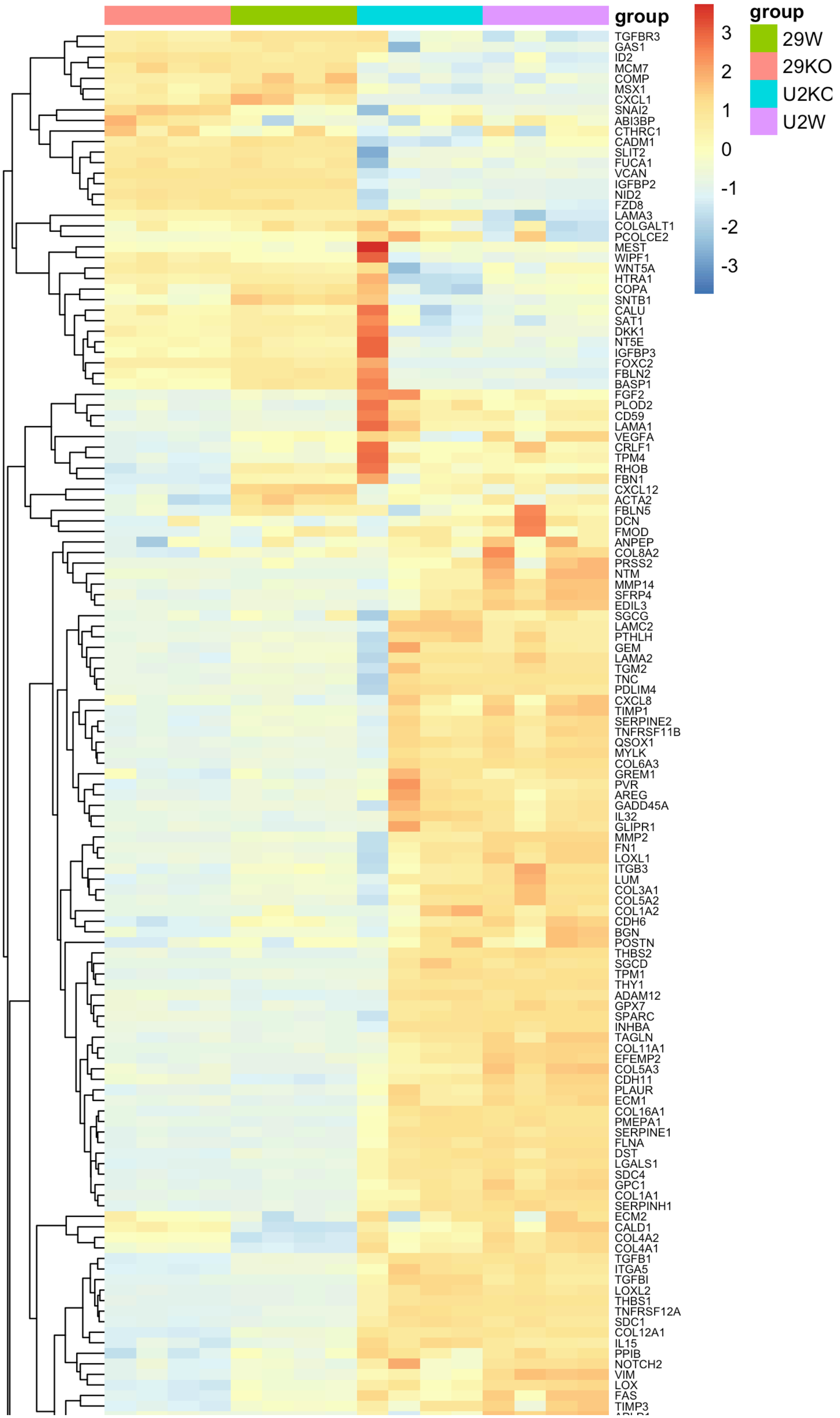
group
TGFBR3
GAS1
ID2
MCM7
COMP
MSX1
CXCL1
SNAI2
ABI3BP
CTHRC1
CADM1
SLIT2
FUCA1
VCAN
IGFBP2
NID2
FZD8
LAMA3
COLGALT1
PCOLCE2
MEST
WIPF1
WNT5A
HTRA1
COPA
SNTB1
CALU
SAT1
DKK1
NT5E
IGFBP3
FOXC2
FBLN2
BASP1
FGF2
PLOD2
CD59
LAMA1
VEGFA
CRLF1
TPM4
RHOB
FBN1
CXCL12
ACTA2
FBLN5
DCN
FMOD
ANPEP
COL8A2
PRSS2
NTM
MMP14
SFRP4
EDIL3
SGCG
LAMC2
PTHLH
GEM
LAMA2
TGM2
TNC
PDLIM4
CXCL8
TIMP1
SERPINE2
TNFRSF11B
QSOX1
MYLK
COL6A3
GREM1
PVR
AREG
GADD45A
IL32
GLIPR1
MMP2
FN1
LOXL1
ITGB3
LUM
COL3A1
COL5A2
COL1A2
CDH6
BGN
POSTN
THBS2
SGCD
TPM1
THY1
ADAM12
GPX7
SPARC
INHBA
TAGLN
COL11A1
EFEMP2
COL5A3
CDH11
PLAUR
ECM1
COL16A1
PMEPA1
SERPINE1
FLNA
DST
LGALS1
SDC4
GPC1
COL1A1
SERPINH1
ECM2
CALD1
COL4A2
COL4A1
TGFB1
ITGA5
TGFBI
LOXL2
THBS1
TNFRSF12A
SDC1
COL12A1
IL15
PPIB
NOTCH2
VIM
LOX
FAS
TIMP3
3
2
1
0
-1
-2
-3
group
29W
29KO
U2KC
U2W

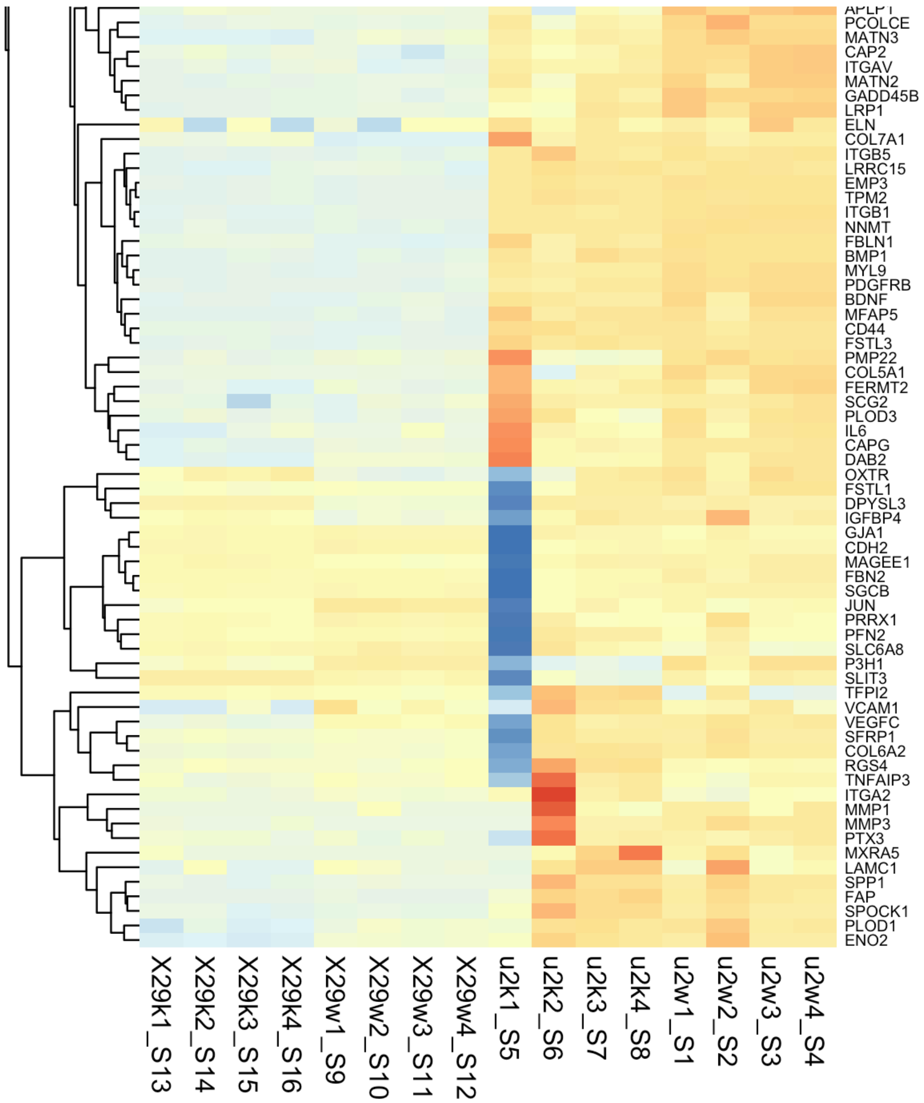

Pathway dataset : MSigDB C2 dataset

CP represents Canonical pathways dataset

Hide

```
msigdb<- msigdbr(species = "Homo sapiens", category = "C2") %>% dplyr::select(gs_name, gene_symbol)
```

Hide

```
ewp <- GSEA(ranks_sorted, TERM2GENE=msigdb)
```

```
using 'fgsea' for GSEA analysis, please cite Korotkevich et al (2019).

preparing geneSet collections...
GSEA analysis...
Warning in preparePathwaysAndStats(pathways, stats, minSize, maxSize, gseaParam,  :
  There are ties in the preranked stats (0.06% of the list).
The order of those tied genes will be arbitrary, which may produce unexpected results.
Warning in fgseaMultilevel(pathways = pathways, stats = stats, minSize = minSize,  :
  For some pathways, in reality P-values are less than 1e-10. You can set the `eps` argument to zero for better e
stimation.
leading edge analysis...
done...
```

Hide

```
head (ewp@result)
```

	ID<br><chr>
SENESE_HDAC1_AND_HDAC2_TARGETS_DN	SENESE_HDAC1_AND_HDAC2_TARGETS_DN

	ID <chr>
KORKOLA_SEMINOMA_UP	KORKOLA_SEMINOMA_UP
SENESE_HDAC2_TARGETS_DN	SENESE_HDAC2_TARGETS_DN
SCHUHMACHER_MYC_TARGETS_UP	SCHUHMACHER_MYC_TARGETS_UP
NIKOLSKY_BREAST_CANCER_16Q24_AMPLICON	NIKOLSKY_BREAST_CANCER_16Q24_AMPLICON
SENESE_HDAC1_TARGETS_DN	SENESE_HDAC1_TARGETS_DN

6 rows | 1-2 of 11 columns

Hide

```
fgseaResTidy <- ewp %>%
  as_tibble() %>%
  arrange(desc(NES))


fgseaResTidy %>%
  dplyr::select(-Description) %>%
  arrange(p.adjust) %>%
  DT::datatable()
```

Show 10 entries Search:

	ID	setSize	enrichmentScore	NES	pvalue	p.ad
1	KORKOLA_SEMINOMA_UP	40	0.8109969353241866	2.711898845635912	1e-10	2.39045454545
2	SCHUHMACHER_MYC_TARGETS_UP	78	0.6862563535836488	2.577918746679877	1e-10	2.39045454545
3	REACTOME_RRNA_PROCESSING	200	0.5597307617371068	2.401006629198304	1e-10	2.39045454545
4	MANALO_HYPOXIA_DN	281	0.5325123810378141	2.370930581144296	1e-10	2.39045454545
5	CHARAFE_BREAST_CANCER_BASAL_VS_MESENCHYMAL_UP	109	0.6001676445638116	2.363345911171792	1e-10	2.39045454545
6	ONDER_CDH1_TARGETS_2_DN	419	0.5024088707133434	2.31890940045907	1e-10	2.39045454545
7	REACTOME_TRANSLATION	274	0.5131179791836963	2.279425451915317	1e-10	2.39045454545
8	MUELLER_PLURINET	288	0.5055720026803664	2.255325823971478	1e-10	2.39045454545
9	BENPORATH_ES_1	352	0.4689359469635439	2.142313968055586	1e-10	2.39045454545
10	GARY_CD5_TARGETS_DN	430	0.4572222956085397	2.118516990376988	1e-10	2.39045454545

Showing 1 to 10 of 473 entries

Previous 1 2 3 4 5 … 48 Next

Hide

```
library(dplyr)
```

Hide

```
ewp2 <- arrange(ewp, desc(abs(NES))) %>% group_by(sign(NES))
```

Hide

```
library(ggplotify)
library(ggplot2)
library(enrichplot)
library(forcats)
```

Hide

```
ggplot(ewp2, showCategory=20,aes(NES, fct_reorder(Description, NES),fill=qvalue)) + geom_col() +
scale_fill_gradientn(colours=c("#b3eebe", "#46bac2", "#371ea3"),
guide=guide_colorbar(reverse=TRUE))+
xlab("Normalized Enrichment Score") + ylab(NULL) +
  ggtitle("canonical pathways")
```

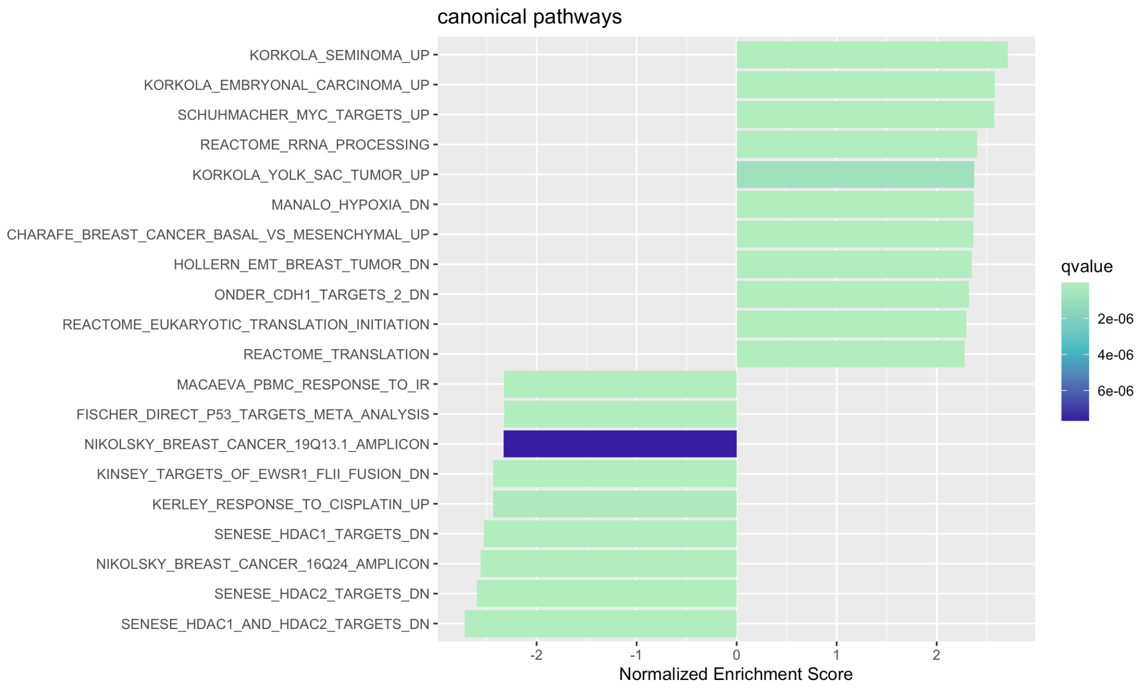

Hide

```
ewp3 <- arrange(ewp, desc(abs(NES)))
```

visualisation using clusterprofiler

Hide

```
## visualization
color <- c("#f7ca64", "#43a5bf", "#86c697", "#a670d6", "#ef998a","#f7ca64", "#43a5bf", "#86c697", "#a670d6", "#ef
998a")
ech4 <- gseaplot2(ewp3, 1:7, color = color, pvalue_table=F, base_size=10)

ech4
```

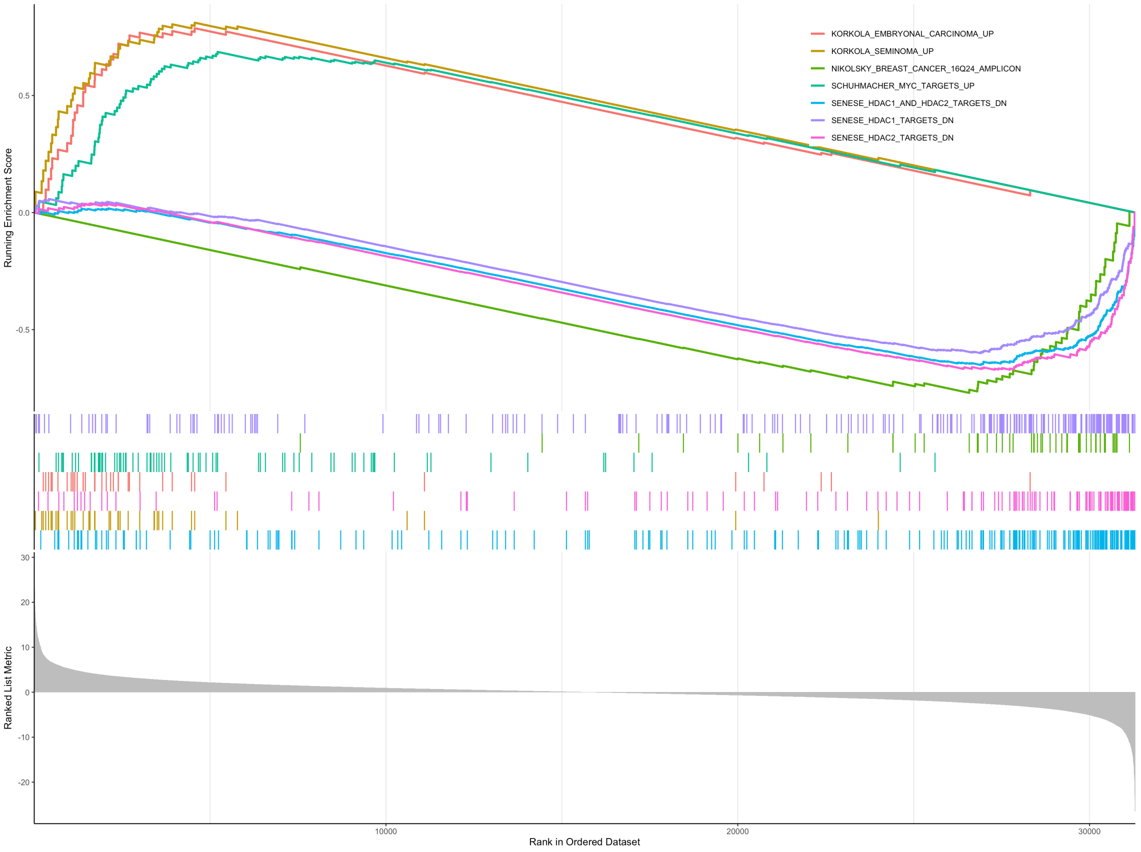

Hide

```
ech3 <- upsetplot(ewp3, n=10)

ech3
```

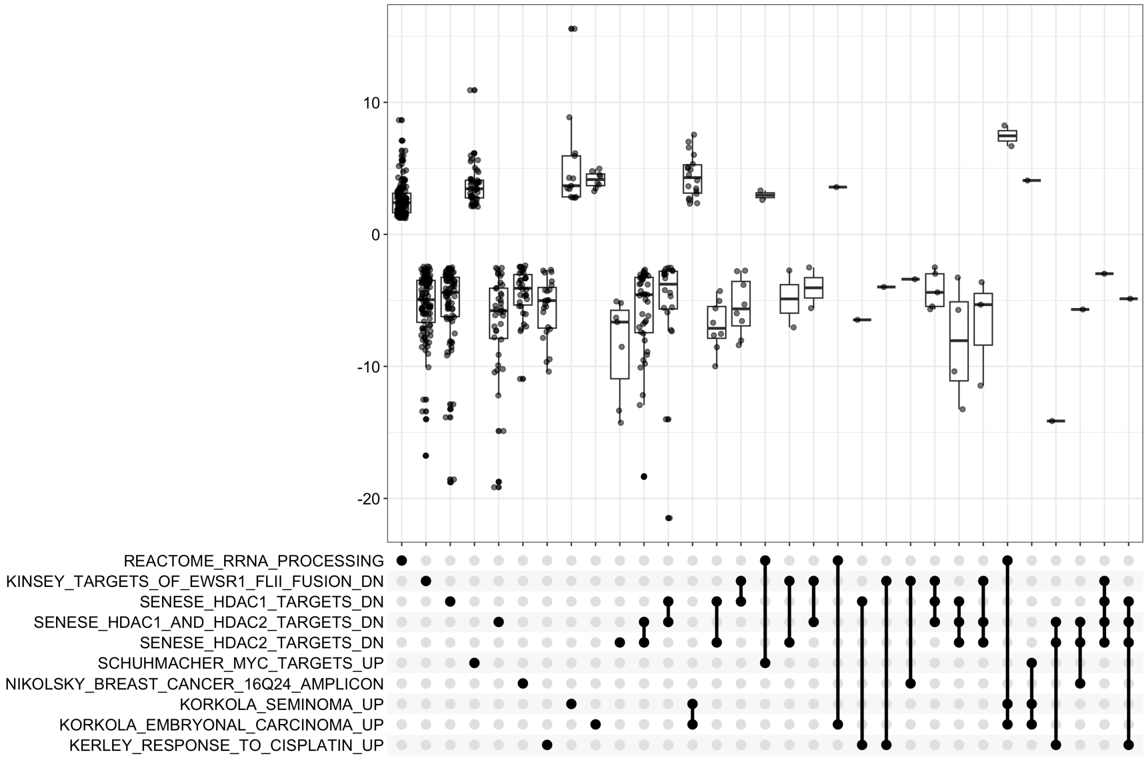

Pathway dataset : MSigDB C5 dataset

C5 represents Ontology gene sets

Hide

```
msigdb<- msigdbr(species = "Homo sapiens", category = "C5") %>% dplyr::select(gs_name, gene_symbol)
```

Hide

```
ewp <- GSEA(ranks_sorted, TERM2GENE=msigdb)
```

```
using 'fgsea' for GSEA analysis, please cite Korotkevich et al (2019).

preparing geneSet collections...
GSEA analysis...
Warning in preparePathwaysAndStats(pathways, stats, minSize, maxSize, gseaParam,  :
  There are ties in the preranked stats (0.06% of the list).
The order of those tied genes will be arbitrary, which may produce unexpected results.
Warning in fgseaMultilevel(pathways = pathways, stats = stats, minSize = minSize,  :
  For some pathways, in reality P-values are less than 1e-10. You can set the `eps` argument to zero for better e
stimation.
leading edge analysis...
done...
```

Hide

```
head (ewp@result)
```

	ID<br><chr>
GOBP_NCRNA_PROCESSING	GOBP_NCRNA_PROCESSING
GOBP_RIBOSOME_BIOGENESIS	GOBP_RIBOSOME_BIOGENESIS
GOBP_RIBONUCLEOPROTEIN_COMPLEX_BIOGENESIS	GOBP_RIBONUCLEOPROTEIN_COMPLEX_BIOGENESIS
GOBP_RRNA_METABOLIC_PROCESS	GOBP_RRNA_METABOLIC_PROCESS
GOBP_NCRNA_METABOLIC_PROCESS	GOBP_NCRNA_METABOLIC_PROCESS
GOCC_MITOCHONDRIAL_PROTEIN_CONTAINING_COMPLEX	GOCC_MITOCHONDRIAL_PROTEIN_CONTAINING_COMPLEX

6 rows | 1-2 of 11 columns

Hide

```
fgseaResTidy <- ewp %>%
  as_tibble() %>%
  arrange(desc(NES))


fgseaResTidy %>%
  dplyr::select(-Description) %>%
  arrange(p.adjust) %>%
  DT::datatable()
```

Show 10 **entries** **Search:**

	ID	setSize	enrichmentScore	NES	pvalue	p.adjust	
1	GOBP_NCRNA_PROCESSING	370	0.5437353289309486	2.487088558246221	1e-10	8.4025e-8	7.1
2	GOBP_RIBOSOME_BIOGENESIS	294	0.555795705204646	2.472183010872091	1e-10	8.4025e-8	7.1
3	GOBP_RIBONUCLEOPROTEIN_COMPLEX_BIOGENESIS	425	0.5225786619138194	2.413148766744065	1e-10	8.4025e-8	7.1
4	GOBP_RRNA_METABOLIC_PROCESS	252	0.5341540228226006	2.34801945867076	1e-10	8.4025e-8	7.1
5	GOBP_NCRNA_METABOLIC_PROCESS	486	0.4756000574107058	2.238217482205955	1e-10	8.4025e-8	7.1
6	GOCC_MITOCHONDRIAL_PROTEIN_CONTAINING_COMPLEX	252	0.5035368822854043	2.213433480322583	1e-10	8.4025e-8	7.1

	ID	setSize	enrichmentScore	NES	pvalue	p.adjust	
7	GOCC_RIBOSOME	208	0.5137268834488169	2.193197338697982	1e-10	8.4025e-8	7.1
8	GOCC_RIBOSOMAL_SUBUNIT	175	0.5240410165601945	2.1923068522974	1e-10	8.4025e-8	7.1
9	GOMF_CATALYTIC_ACTIVITY_ACTING_ON_RNA	354	0.4453208829067836	2.032028626139846	1e-10	8.4025e-8	7.1
10	GOCC_ORGANELLE_INNER_MEMBRANE	496	0.4022637783484048	1.896777576533008	1e-10	8.4025e-8	7.1

Showing 1 to 10 of 443 entries

Previous 1 2 3 4 5 … 45 Next

Hide

```
library(dplyr)
```

Hide

```
ewp2 <- arrange(ewp, desc(abs(NES))) %>% group_by(sign(NES))
```

Hide

```
library(ggplotify)
library(ggplot2)
library(enrichplot)
library(forcats)
```

Hide

```
ggplot(ewp2, showCategory=20,aes(NES, fct_reorder(Description, NES),fill=qvalue)) + geom_col() +
scale_fill_gradientn(colours=c("#b3eebe", "#46bac2", "#371ea3"),
guide=guide_colorbar(reverse=TRUE))+
xlab("Normalized Enrichment Score") + ylab(NULL) +
  ggtitle("C5 pathways")
```

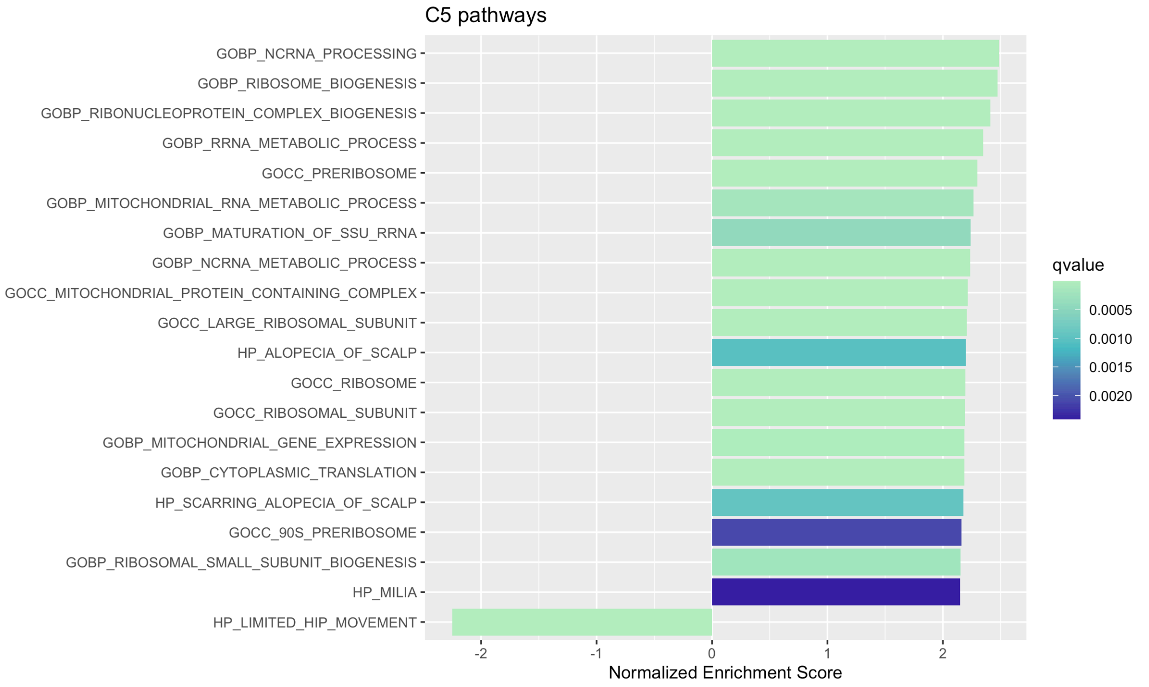

Hide

```
ewp3 <- arrange(ewp, desc(abs(NES)))
```

visualisation using clusterprofiler

Hide

```
## visualization
color <- c("#f7ca64", "#43a5bf", "#86c697", "#a670d6", "#ef998a","#f7ca64", "#43a5bf", "#86c697", "#a670d6", "#ef
998a")
ech4 <- gseaplot2(ewp3, 1:10, color = color, pvalue_table=F, base_size=10)

ech4
```

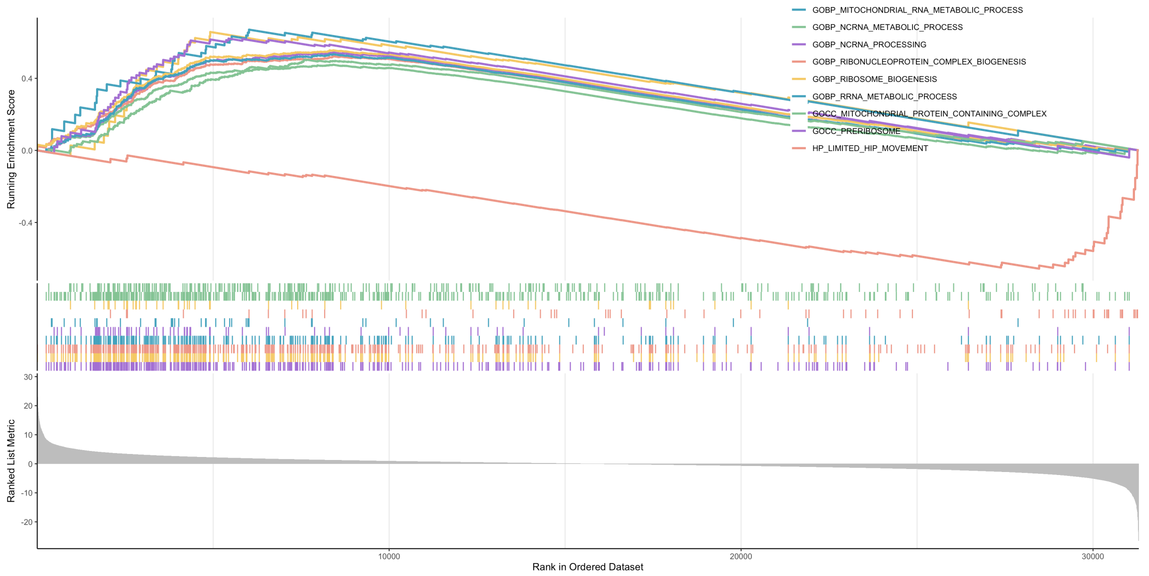

Hide

```
ech3 <- upsetplot(ewp3, n=10)

ech3
```

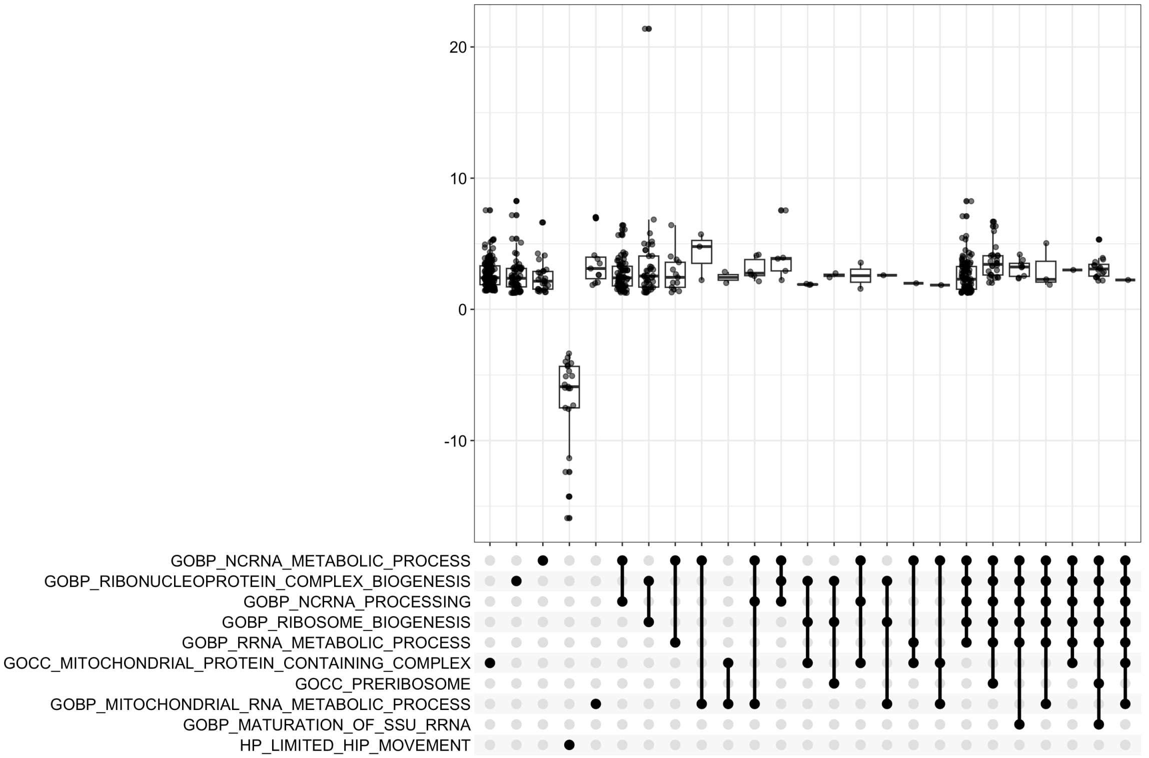

Hide

```
sessionInfo()
```

```
R version 4.4.0 (2024-04-24)
Platform: aarch64-apple-darwin20
Running under: macOS Ventura 13.0

Matrix products: default
BLAS:   /System/Library/Frameworks/Accelerate.framework/Versions/A/Frameworks/vecLib.framework/Versions/A/libBLA
S.dylib
LAPACK: /Library/Frameworks/R.framework/Versions/4.4-arm64/Resources/lib/libRlapack.dylib;  LAPACK version 3.12.0

locale:
[1] en_US.UTF-8/en_US.UTF-8/en_US.UTF-8/C/en_US.UTF-8/en_US.UTF-8

time zone: America/New_York
tzcode source: internal

attached base packages:
[1] grid      stats4    stats     graphics  grDevices utils     datasets  methods   base

other attached packages:
 [1] org.Hs.eg.db_3.19.1         GO.db_3.19.1                AnnotationDbi_1.66.0
 [4] fgsea_1.30.0                xlsx_0.6.5                  readxl_1.4.3
 [7] VennDiagram_1.7.3           futile.logger_1.4.3         ggvenn_0.1.10
[10] ggpubr_0.6.0                reshape2_1.4.4              pheatmap_1.0.12
[13] Rgraphviz_2.48.0            graph_1.82.0                biomaRt_2.60.1
[16] ggfortify_0.4.17            msigdbr_7.5.1               enrichplot_1.24.0
[19] ggplotify_0.1.2             clusterProfiler_4.12.0      ggrepel_0.9.5
[22] lubridate_1.9.3             forcats_1.0.0               stringr_1.5.1
[25] dplyr_1.1.4                 purrr_1.0.2                 readr_2.1.5
[28] tidyr_1.3.1                 tibble_3.2.1                ggplot2_3.5.1
[31] tidyverse_2.0.0             DESeq2_1.44.0               SummarizedExperiment_1.34.0
[34] Biobase_2.64.0              MatrixGenerics_1.16.0       matrixStats_1.3.0
[37] GenomicRanges_1.56.1        GenomeInfoDb_1.40.1         IRanges_2.38.1
[40] S4Vectors_0.42.1            BiocGenerics_0.50.0         apeglm_1.26.1

loaded via a namespace (and not attached):
  [1] splines_4.4.0           filelock_1.0.3          cellranger_1.1.0        polyclip_1.10-6
  [5] lifecycle_1.0.4         httr2_1.0.2             rstatix_0.7.2           lattice_0.22-6
  [9] MASS_7.3-61             crosstalk_1.2.1         backports_1.5.0         magrittr_2.0.3
 [13] sass_0.4.9              rmarkdown_2.27          jquerylib_0.1.4         yaml_2.3.9
 [17] cowplot_1.1.3           DBI_1.2.3               RColorBrewer_1.1-3      abind_1.4-5
 [21] pkgload_1.4.0           zlibbioc_1.50.0         ggraph_2.2.1            yulab.utils_0.1.4
 [25] xlsxjars_0.6.1          tweenr_2.0.3            rappdirs_0.3.3          GenomeInfoDbData_1.2.12
 [29] tidytree_0.4.6          codetools_0.2-20        DelayedArray_0.30.1     DOSE_3.30.1
 [33] DT_0.33                 xml2_1.3.6              ggforce_0.4.2           tidyselect_1.2.1
 [37] aplot_0.2.3             UCSC.utils_1.0.0        farver_2.1.2            viridis_0.6.5
 [41] BiocFileCache_2.12.0    jsonlite_1.8.8          Formula_1.2-5           tidygraph_1.3.1
 [45] systemfonts_1.1.0       bbmle_1.0.25.1          tools_4.4.0             progress_1.2.3
 [49] ragg_1.3.2              treeio_1.28.0           Rcpp_1.0.13             glue_1.7.0
 [53] gridExtra_2.3           SparseArray_1.4.8       xfun_0.45               qvalue_2.36.0
 [57] withr_3.0.0             numDeriv_2016.8-1.1     formatR_1.14            fastmap_1.2.0
 [61] fansi_1.0.6             digest_0.6.36           timechange_0.3.0        R6_2.5.1
 [65] gridGraphics_0.5-1      textshaping_0.4.0       colorspace_2.1-0        RSQLite_2.3.7
 [69] utf8_1.2.4              generics_0.1.3          data.table_1.15.4       prettyunits_1.2.0
 [73] graphlayouts_1.1.1      httr_1.4.7              htmlwidgets_1.6.4       S4Arrays_1.4.1
 [77] scatterpie_0.2.3        pkgconfig_2.0.3         rJava_1.0-11            gtable_0.3.5
 [81] blob_1.2.4              XVector_0.44.0          shadowtext_0.1.3        htmltools_0.5.8.1
 [85] carData_3.0-5           scales_1.3.0            ggupset_0.4.0           png_0.1-8
 [89] ggfun_0.1.5             lambda.r_1.2.4          knitr_1.48              rstudioapi_0.16.0
 [93] tzdb_0.4.0              coda_0.19-4.1           nlme_3.1-165            curl_5.2.1
 [97] bdsmatrix_1.3-7         cachem_1.1.0            parallel_4.4.0          HDO.db_0.99.1
[101] pillar_1.9.0            vctrs_0.6.5             car_3.1-3               dbplyr_2.5.0
[105] evaluate_0.24.0         futile.options_1.0.1    mvtnorm_1.2-5           cli_3.6.3
[109] locfit_1.5-9.10         compiler_4.4.0          rlang_1.1.4             crayon_1.5.3
[113] ggsignif_0.6.4          labeling_0.4.3          emdbook_1.3.13          plyr_1.8.9
[117] fs_1.6.4                stringi_1.8.4           viridisLite_0.4.2       BiocParallel_1.38.0
[121] babelgene_22.9          munsell_0.5.1           Biostrings_2.72.1       lazyeval_0.2.2
[125] GOSemSim_2.30.0         Matrix_1.7-0            hms_1.1.3               patchwork_1.2.0
[129] bit64_4.0.5             KEGGREST_1.44.1         igraph_2.0.3            broom_1.0.6
[133] memoise_2.0.1           bslib_0.7.0             ggtree_3.12.0           fastmatch_1.1-4
[137] bit_4.0.5               ape_5.8                 gson_0.1.0
```

Add a new chunk by clicking the *Insert Chunk* button on the toolbar or by pressing *Cmd+Option+I*.

When you save the notebook, an HTML file containing the code and output will be saved alongside it (click the *Preview* button or press *Cmd+Shift+K* to preview the HTML file).

The preview shows you a rendered HTML copy of the contents of the editor. Consequently, unlike *Knit*, *Preview* does not run any R code chunks. Instead, the output of the chunk when it was last run in the editor is displayed.
